# Prostaglandin-based rAAV-mediated glaucoma gene therapy in Brown Norway rats

**DOI:** 10.1038/s42003-022-04134-w

**Published:** 2022-11-03

**Authors:** Kristina J. Chern, Emily R. Nettesheim, Christopher A. Reid, Nathan W. Li, Gavin J. Marcoe, Daniel M. Lipinski

**Affiliations:** 1 Department of Cell Biology, Neurobiology and Anatomy, Medical College of Wisconsin, Milwaukee, WI USA; 2 Department of Ophthalmology and Visual Sciences, Medical College of Wisconsin, Milwaukee, WI USA

**Keywords:** Molecular medicine, Gene therapy

## Abstract

Prostaglandin analogs are first-line treatments for open angle glaucoma and while effective at lowering intraocular pressure, they are undermined by patient non-compliance, causing atrophy of the optic nerve and severe visual impairment. Herein, we evaluate the safety and efficacy of a recombinant adeno-associated viral vector-mediated gene therapy aimed at permanently lowering intraocular pressure through *de novo* biosynthesis of prostaglandin F2α within the anterior chamber. This study demonstrated a dose dependent reduction in intraocular pressure in normotensive Brown Norway rats maintained over 12-months. Crucially, therapy could be temporarily halted through off-type riboswitch activation, reverting intraocular pressure to normal. Longitudinal multimodal imaging, electrophysiology, and post-mortem histology revealed the therapy was well tolerated at low and medium doses, with no major adverse effects to anterior chamber health, offering a promising alternative to current treatment strategies leading to clinically relevant reductions in intraocular pressure without the need for adherence to a daily treatment regimen.

## Abstract (web summary)

A well-tolerated rAAV vector-mediated gene therapy in normotensive Brown Norway rats is used to reduce intraocular pressure in a dose dependent manner, encouraging further evaluations on pre-clinical models of glaucoma.

## Introduction

Open angle glaucoma (OAG) is a complex ocular disorder affecting approximately 79.6 million individuals worldwide resulting in progressive loss of retinal ganglion cells, axonal damage within the optic nerve, and ultimately, blindness^1^. While open angle glaucoma has multiple environmental and genetic risk factors, the physiopathology underlying IOP elevation centers on the imbalance between aqueous humor production by the ciliary body and aqueous drainage through the trabecular meshwork^2^. As a consequence, current treatment strategies—both pharmacological and surgical—focus on achieving a reduction in IOP through either inhibition of aqueous production or increasing aqueous drainage, with the goal of lessening mechanical pressure on the retina and optic nerve in order to preserve vision^3–5^.

Pharmacological agents are administered as first line treatments and fall into five main classes—carbonic anhydrase inhibitors, adrenergic agonists, RHO kinase inhibitors, beta-blockers, and prostaglandin F2α (PGF_2α_) analogs—with the latter being most widely utilized^6–8^. PGF_2α_ analogs, such as Latanoprost, Bimatoprost, and Travoprost, are applied topically as pro-drugs to the corneal surface via eye drops, where they are absorbed across the corneal epithelium and hydrolyzed into active conformations as they traverse the stroma, before being released into the aqueous humor. Following release into the aqueous humor, PGF_2α_ analogs lead to increased drainage through the uveoscleral outflow pathway following remodeling of the extracellular matrix surrounding the ciliary muscle^6,9–13^. While the use of topical agents can be effective, leading to an IOP reduction of approximately 25%, their use is associated with numerous side effects, including ocular surface irritation, blurred vision, iris discoloration (hyperpigmentation), redness (hyperemia), excessive growth or thickening of the eye lashes (hypertrichosis), deepening of the eye lids (orbitopathy), hyper-sensitivity to light, and recurrence of concurrent infections (e.g., herpetic keratitis)^14–18^. As a result of the poor tolerability of current pharmacological treatments for OAG, coupled with difficulties associated with correctly applying eye drops and the lack of immediate negative feedback when doses are missed, patients often struggle with adherence to daily treatment regimens. Indeed, studies where patient eye drop usage was electronically monitored demonstrated extremely poor compliance with existing pharmacological treatments, with 44% of patients using their eye drops less than 75% of the time^19,20^.

Consequently, in recent years there has been a drive to develop longer acting pharmacological treatments with a focus on sustained release drug implants, which have been studied both in large animal and human clinical trials^21–26^. Implants, such as sustained released Bimatoprost, have been demonstrated to cause a reduction in IOP for up to 24 months; however, only 28% of participants maintained a controlled IOP over that period and a further 26.5% of patients needed rescue eye drops or re-administration of implants following degradation^23^. In addition to achieving a sustained reduction in IOP in the majority of patients, one other notable benefit of such implants is that the incidence of certain side effects was reduced relative to patients receiving topical eye drops, though patients receiving sustained release Bimatoprost did exhibit conjunctival hyperemia, macular edema and intraocular inflammation^27^. While implants have proven to be relatively safe, there are patients who develop corneal endothelial cell loss as a result of implant replacement and as such the Federal Drug Administration (FDA) has so far limited patients to one 10 µg implant per eye with no re-administration permitted^27^.

Despite the genetic etiology underlying the development of OAG being incompletely understood in the majority of patients, gene therapy remains an attractive therapeutic strategy that may allow for life-long correction of the disease phenotype following a single intervention^28–30^. Owing to the unique properties of the eye, ophthalmic indications have been at the forefront of the gene therapy field over the past several decades and have typically focused on the use of recombinant adeno-associated virus (rAAV) vectors, which have been shown in numerous pre-clinical and clinical studies to mediate safe and persistent transgene expression in a variety of ocular cell types and across a range of species^31–35^. While the majority of studies focused on the treatment of rare inherited monogenic retinal disorders, such as Leber’s congenital amaurosis, choroideremia, and achromatopsia, gene therapy may also be used to treat diseases with complex etiology, such as OAG, so long as the underlying physiopathology is well understood^36–42^. As decades of clinical practice have demonstrated a clear correlation between IOP reduction and positive visual outcomes in patients with OAG, we propose that ocular hypertension represents a clear and modifiable risk factor that could be modulated via a gene therapy approach in order to preserve vision in OAG patients. Specifically, as prostaglandin analogs are a gold standard for OAG treatment and are highly effective at reducing IOP when not undermined by poor patient compliance, we aimed to develop a gene therapy treatment to drive biosynthesis of PGF_2α_ directly within the eye through rAAV-mediated over-expression of prostaglandin-endoperoxide synthase 2 (*COX2*)—the rate limiting enzyme in the biosynthesis of PGF_2α_—from within cells of the anterior chamber^43^. We hypothesize the development of a rAAV-mediated gene therapy to synthesize and release native PGF_2α_ may be sufficient to lower IOP in a safe and sustained manner, leading to improved visual outcomes through the elimination of patient non-compliance whilst also reducing the incidence and severity of the side effects observed when prostaglandin analogs are administered topically.

Herein, we utilize normotensive Brown Norway (BN) rats to evaluate the safety and efficacy of our PGF_2α_ expressing rAAV gene therapy treatment aimed at lowering IOP in patients suffering from ocular hypertension following a single therapeutic dose. Using a combination of multimodal imaging, electrophysiology, tonometry, and post-mortem histology, we demonstrate a clear dose-dependent relationship between vector genomes administered and IOP. Significantly, we achieved a clinically relevant reduction in ocular hypertension for a period of 12-months at a well-tolerated dose. Uniquely, through the inclusion of tetracycline-responsive riboswitch elements within the expression cassette, we demonstrate that transgene expression can be temporarily, but effectively, ‘switched off’ through administration of an oral drug, leading to a reversion towards normotension. This therapy would represent a paradigm shift in the clinical management of OAG, allowing for improved visual outcomes by removing patient compliance issues, and increasing patient quality of life through decreased medical burden, costs, and reduced side effects.

## Results

### rAAV-mediated over-expression of *COX2* and *PTGFR* leads to a dose-dependent reduction in IOP that is sustained over a period of 12-months and can be partially reversed

In order to facilitate *de novo* production of PGF_2α_ from cells of the anterior chamber, we first cloned an expression cassette encoding codon optimized human *COX2*—the rate limiting enzyme in PGF_2α_ biosynthesis—in addition to the human prostaglandin F receptor (*PTGFR*) separated by a P2A cleavage site and driven by a ubiquitously expressing small chicken beta actin (CBA) promoter. Inclusion of *PTGFR* within the expression construct was chosen in order to build a ‘biological circuit’ wherein both the effector molecule (PGF_2α_) and its own receptor are present at high levels within the target tissue, a strategy that has been shown previously in cats to lead to greater reduction in IOP than the expression of *COX2* alone^44^.

The expression cassette is flanked by inverted terminal repeats (ITRs) derived from AAV2 to allow packaging into a recombinant rAAV vector, and also contains two ‘off-type’ tetracycline-responsive riboswitch elements (TC40 and TC45), which we have demonstrated previously allow for post-transcriptional down-regulation of transgene expression from a rAAV vector injected into the eye upon oral dosing of the activating ligand (i.e., tetracycline)^45,46^. While the TC40 and TC45 riboswitches have not been expressed in cells of the anterior chamber, it is known that tetracycline accumulates within the cornea and aqueous humor following oral dosing and as such, is expected to be bioavailable to the riboswitch elements to drive gene modulation^47^. The inclusion of an off-switch in the expression cassette is a critical consideration for the development of any prostaglandin-based IOP lowering gene therapy treatment, where clinical management of OAG can necessitate temporary cessation of PGF_2α_ treatment in the event of a patient developing a concurrent infection, such as herpetic keratitis^18,48,49^.

**Fig. 1:**
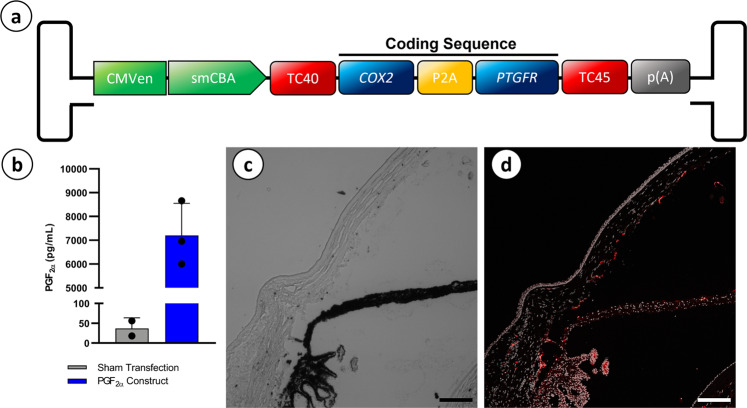
Construct schematic and confirmation of in vitro activity and in vivo tropism. A diagrammatical representation of the CCPP transgene driven by a CMV enhancer and smCBA promotor while containing two tetracycline off-type riboswitches (TC40 and TC45), as well as human codon optimized *COX2* and *PTGFR* separated by a P2A cleavage signal (**a**). A PGF_2*ɑ*_ ELISA confirmed significantly higher concentrations of PGF_2*ɑ*_ in cell culture media after transfection with CCPP transgene than media collected from sham transfected cells (**b**) (unpaired T-test, *p* = 0.0056, *N* = 2 (sham transfected) *N* = 3 (CCPP transfected), error bars = SD). Brightfield and fluorescent imaging of rAAV2/2[MAX]-smCBA-mCherry eyes 8-weeks post injection confirm vector tropism within the iris, corneal endothelium, and ciliary body (**c**, **d**). Scale bar = 150 µm, Magnification = 20×.

The biological activity of the resulting CBA.*COX2*.P2A.*PTGFR*.TC40/TC45 construct (termed herein, CCPP) (Fig. 1a) was initially validated in vitro by transfection of HEK293T cells, resulting in significantly increased (unpaired T-test, *p* = 0.0056, *N* = 2 (sham transfected), *N* = 3 (CCPP transfected)) levels of PGF_2α_ being secreted into the culture media as compared to sham transfected controls (Fig. 1b). Having established that the CCPP expression cassette catalyzes significantly increased production of PGF_2α,_ the construct was subsequently packaged into rAAV2/2[MAX], an rAAV2-derived capsid mutant vector containing multiple single point mutations (Quad YF + TV: Y272F, Y444F, Y500F, Y730F, and T491V) and a peptide insertion (7m8: R588_Q589insLALGETTRPA) known to increase transduction efficiency and tissue penetrance in several ocular cell types^50–52^. When injected into the anterior chamber of BN rats this capsid mutant serotype vector transduces cells of the peripheral corneal endothelium, iris, and iridocorneal angle (Fig. 1c, d).

Juvenile (post-natal week 6–8) BN rats (*N* = 30: 15 male and 15 female) imported from a commercial breeder underwent comprehensive ophthalmological examinations to exclude the presence of developmental or other abnormalities in retinal or corneal structure or function. Importantly, all animals exhibited stable intraocular pressure (IOP; 19.52 ± 3.23 mmHg, 1 SD, *N* = 30 eyes) prior to receiving intra-ocular rAAV2/2[MAX].CCPP injections. BN rats were assigned randomly to one of three experimental cohorts and received a unilateral intracameral injection of equal volume (10 μl) containing either a low (3.9 × 10^9^ vg/eye), medium (3.9 × 10^10^ vg/eye) or high (3.9 × 10^11^ vg/eye) dose of purified rAAV2/2[MAX].CCPP vector. The contralateral eye in all animals remained uninjected to serve as an intra-animal control and to limit any vector reflux that may occur during repositioning of the animal.

**Fig. 2:**
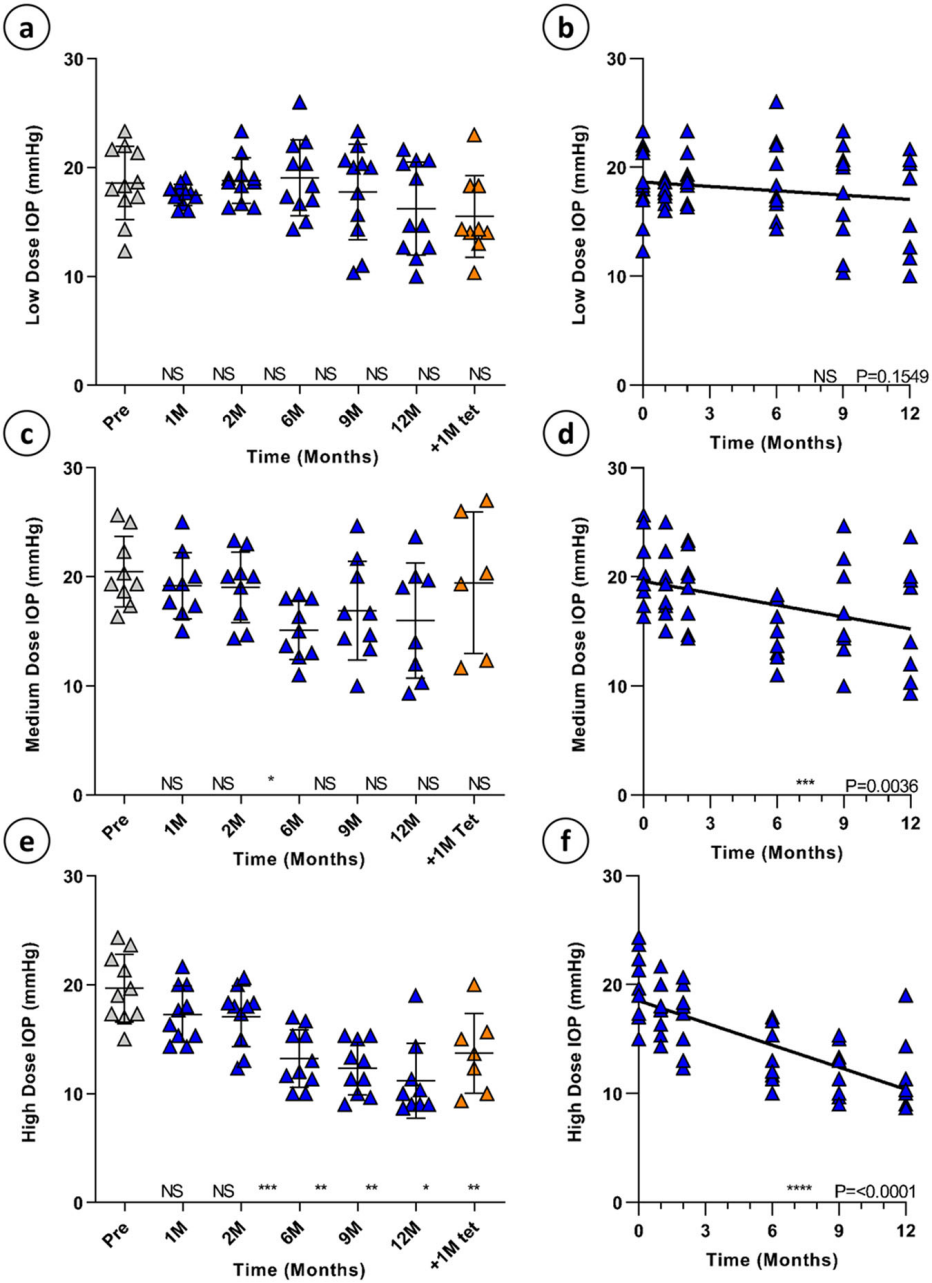
Transgene expression’s effect on intraocular pressure. A dose dependent reduction in IOP was found between groups: low, medium, and high dose animals showed a reduction of 12.6%, 21.87%, and 43.2% at 12 months respectively (**a**, **c**, and **e**) that was maintained over 12 months (**b**; simple linear regression, *p* = 0.1549, *N* = 11 **d**; *p* = 0.0036, *N* = 9 and **f**; *p* < 0.0001, *N* = 10, Error bars = SD). At 12 months, animals were administered 5% w/w tetracycline diet and IOP increased by 21.5% and 22.5% in medium and high dose animals respectively after 1 month (**c** and **e**).

At 1-, 2-, 6-, 9- and 12-months post-injection, rebound tonometry was repeated on conscious BN rats by examiners who were masked with respect to the treatment group to enable alterations in IOP in response to therapy to be measured objectively. Low dose BN rats exhibited non-significant (NS) reductions in IOP at all points during the time course compared to baseline measurements (Fig. 2a; Dunnett’s multiple comparison test, *p*  ≥ 0.05 all comparisons, *N* = 11) and no significant trend was observed over the entire 12-month evaluation period (Fig. 2b; simple linear regression, *p* = 0.1549).

Medium dose rats exhibited a significant reduction in IOP at 6 months (Fig. 2c; Dunnett’s multiple comparison test, *p* = 0.01, *N* = 9) and there was a significant trend of decreasing IOP noted over the 12-month period (Fig. 2d; simple linear regression, *p* = 0.0036) with a reduction in IOP of 21.88% from baseline to 12 months.

High dose treated rats displayed a significant reduction from baseline IOP at 6-, 9-, and 12-month time points (Fig. 2e; Dunnett’s multiple comparison test, *p* ≤ 0.0021, *N* = 10) with a highly significant trend of reducing IOP over the 12-month period (Fig. 2f, simple linear regression, *p* < 0.0001). High dose treated rats exhibited the highest reduction in IOP from baseline at 12 months, with an overall decrease of 43.22%. Importantly, no treated eyes in any dosage group at any time point showed evidence of hypotony (IOP ≤ 5 mmHg) throughout the study.

At 12-months post-injection, one male and one female rat from each dosage group was sacrificed for histology, and remaining animals placed on a custom diet containing 5% w/w tetracycline to ‘switch off’ gene expression through activation of the TC40 and TC45 riboswitch elements incorporated within the vector genome. Following one month of tetracycline dietary supplementation, tonometry was repeated to determine whether suppression of transgene expression resulted in a reversion of IOP towards normal tension. BN rats injected with a low dose of the rAAV2/2[MAX].CCPP vector showed no significant changes in IOP compared to either the post-injection tonometry assessments or pre-injection baseline, indicating that tetracycline administration had no direct impact on IOP (Fig. 2a, Dunnett’s multiple comparison test, *p* = 0.4461, *N* = 9). Importantly, IOP of BN rats injected with a medium dose of rAAV2/2[MAX].CCPP vector demonstrated an almost full reversion to normal tension, with IOP increasing 21.5% compared to 12-month measurements (Fig. 2c, *N* = 6). High dose animals showed a significant reversion to baseline IOP (Fig. 2e; Dunnett’s multiple comparison test, *p* = 0.0283, *N* = 7) with mean IOP increasing 22.5% compared to 12-months.

### rAAV-mediated over-expression of *COX2* and *PTGFR* has no effect on retinal morphology and function

To assess whether *de novo* biosynthesis of PGF_2α_ in the anterior chamber has effects on retinal health, we performed confocal scanning laser ophthalmoscopy (cSLO) and optical coherence tomography (OCT) imaging at baseline and 12-months to detect alterations in retinal reflectance and thickness, respectively. cSLO imaging revealed increased near-infrared reflectance (NIR) and a ‘striped’ fundus appearance at 12-months in both untreated (control) and rAAV2/2[MAX].CCPP vector injected eyes compared to baseline in the low (Supplementary Fig. 1a–d), medium (Supplementary Fig. 1e–h) and high (Supplementary Fig. 1i–l) dose groups, suggesting that any alterations in retinal morphology observed occurred independently of treatment or dose and are instead likely related to normal aging.

**Fig. 3:**
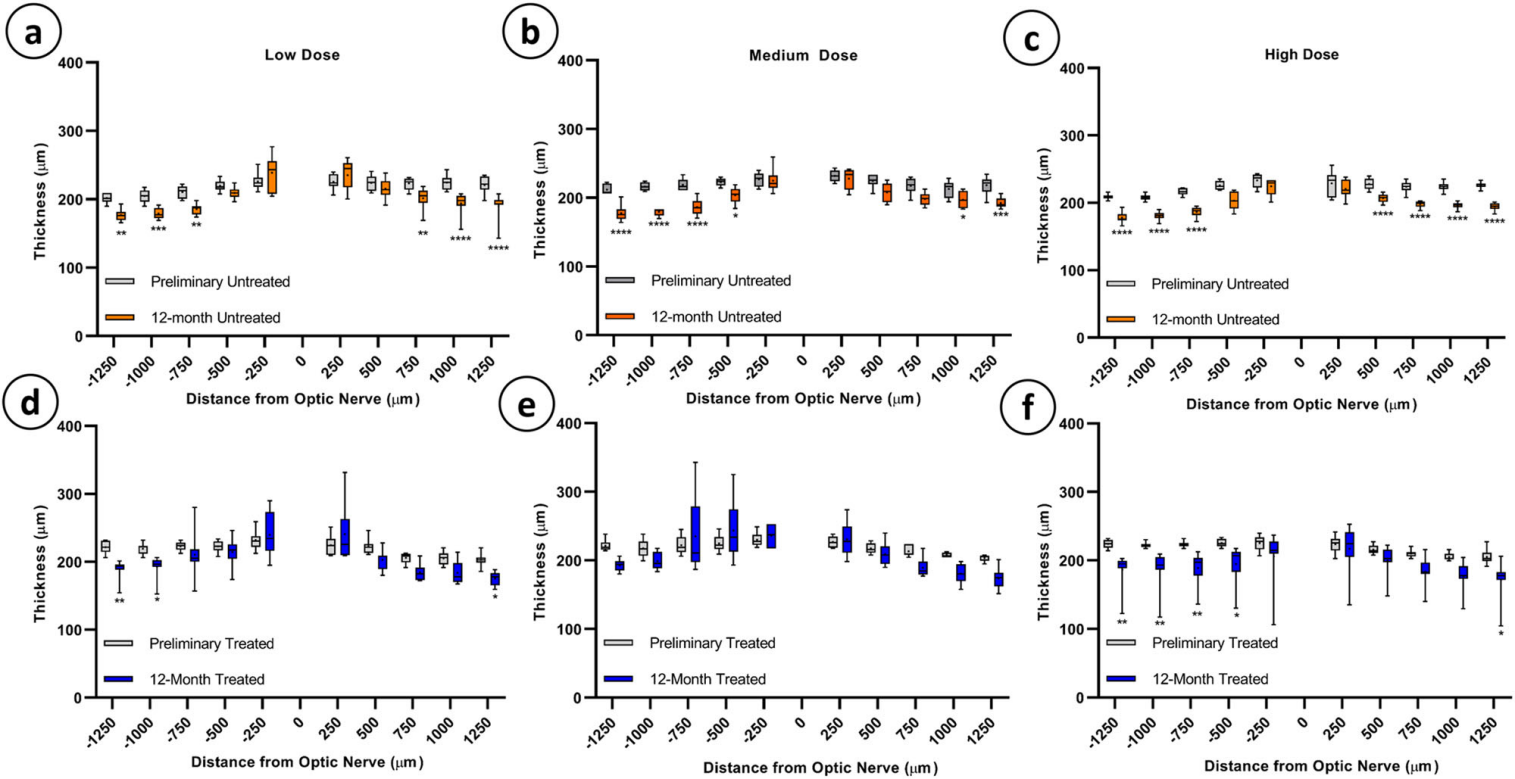
Total retinal thickness at baseline vs. 12-months as assessed by SD-OCT. Significant thinning of the retina was observed at various eccentricities from the optic nerve at the 12-month timepoint for both untreated (**a**–**c**) and treated eyes (**d**, **f**) as compared to baseline measurements. No significant thinning was noted in the medium dose treated eyes (**e**). (Two-way ANOVA with post hoc Sidak’s multiple comparison test, *p* = 0.0123-0.0335 (*), 0.0027-0.0087 (**), 0.0007 (***), and <0.0001(****)). Box plot elements: mean = +, centerline = median, box limits = 25th and 75th percentiles, whiskers = min and max).

Five averaged OCT B-scans through the optic nerve head in all animals at baseline and 12-months post rAAV2/2[MAX].CCPP injection were analyzed in OCT reflectivity analytics (ORA) to quantify retinal thickness (Supplementary Fig. 2)^53^. 10 longitudinal reflectivity profiles (LRPs) were generated at 250-micron increments from the optic nerve and used to determine retinal thickness as measured from the retinal nerve fiber layer through to the RPE (Fig. 3). Significant reduction in retinal thickness was observed at the 12-month timepoint in both untreated (Fig. 3a–c) and rAAV2/2[MAX].CCPP vector injected (Fig. 3d–f) eyes compared to baseline; at least one eccentricity in the high and low dose groups showed significance (two-way ANOVA, Sidak’s multiple comparison test, *p* = 0.0123–0.0335 (*), 0.0027–0.0087 (**), 0.0007 (***), and <0.0001(****), *N* = 8/group), whereas medium-dose rAAV treated eyes (Fig. 3e), showed some degree of thinning, but did not reach significance (two-way ANOVA, Sidak’s multiple comparison test, *p* > 0.1072, *N* = 6). As retinal thinning has been demonstrated in multiple species as a function of age and was observed in treated and untreated eyes in all dose groups, we hypothesize that the observed reduced retinal thickness is also the result of normal aging^54–60^.

**Fig. 4:**
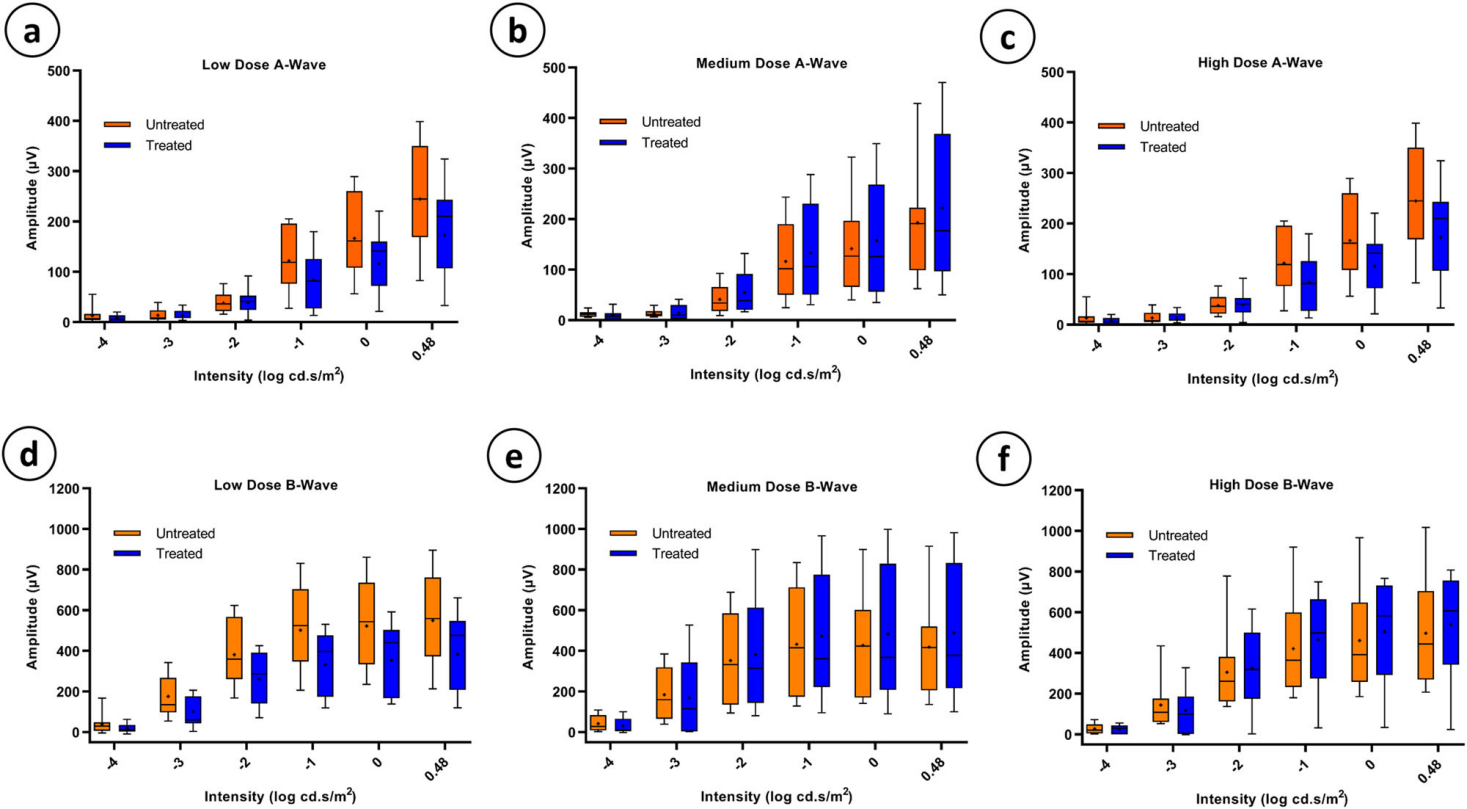
Electroretinography comparison between treated and untreated eyes at 12-months. Quantification of average A and B wave amplitudes from each dosage group are shown (**a**–**f**). No significant changes were found between treated and untreated amplitudes for A or B waves (Multiple T-tests, *p* > 0.05 all groups. *N* = 11, 8, and 10 for low, medium, and high dose respectively. Box plot elements: mean = +, centerline = median, box limits = 25th and 75th percentiles, whiskers = min and max).

To investigate whether CCPP transgene expression has any adverse effect on retinal function, full field electroretinogram (ffERG) recordings were collected for treated and untreated eyes on dark-adapted rats (Fig. 4). No significant differences in A-wave or B-wave amplitudes were found between treated and untreated eyes in low dose (*N* = 11), medium dose (*N* = 8), or high dose groups (*N* = 10) at any intensity (Fig. 4a–f; multiple unpaired T-tests *p* > 0.05 all groups, Supplementary Fig. 3). Despite age-related alterations in retinal appearance and thickness, functional integrity of the retina in both treated and contralateral untreated eyes was maintained over a period of 12-months of ubiquitous CCPP transgene expression.

### rAAV-mediated over-expression of *COX2* and *PTGFR* has no adverse effect on corneal thickness and causes dose-dependent deepening of anterior chamber

**Fig. 5:**
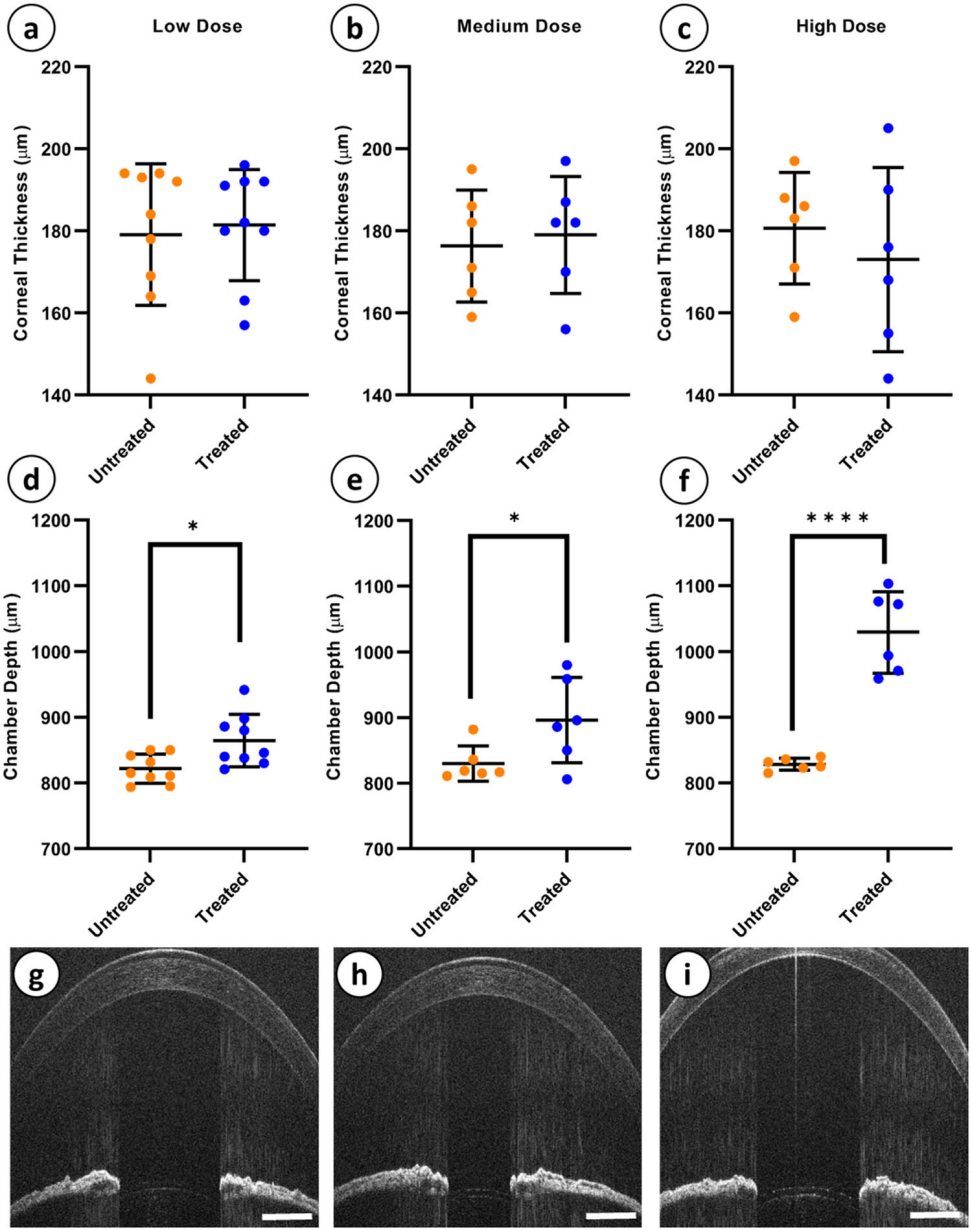
Central corneal thickness and anterior chamber depth of treated and untreated eyes at each dosage. Central corneal thickness was not significantly changed between untreated and treated eyes of any dosage group (**a**–**c**). Anterior chamber depth was found to be significantly increased in low (**d**, **g**), medium (**e**, **h**), and high (**f**, **i**) dose treated eyes as compared to untreated controls. (Unpaired T-test: *p* = 0.7541 (**a**), *p* = 0.0447 (**b**), *p* = 0.2931 (**c**), *p* = 0.0125 (**d**), *p* = 0.0447 (**e**), and *p* < 0.001 (**f**). *N* = 9, 6, and 6, for low, medium, and high dose, respectively. Error bars = SD).

Following intracameral administration of the rAAV2/2[MAX].CCPP vector in BN rats, we sought to investigate the effects of *de novo* PGF_2α_ biosynthesis on anterior chamber health using OCT to determine central corneal thickness and anterior chamber depth. OCT imaging of the cornea conducted at 12-months in rAAV2/2[MAX].CCPP vector injected (treated) and control (untreated) eyes revealed no significant differences in central corneal thickness in either the low (*p* = 0.7541, *N* = 9), medium (*p* = 0.7475, *N* = 6) or high (*p* = 0.2931, *N* = 6,) dose treated eye (Fig. 5a–c; all comparisons unpaired T-tests). By contrast, OCT imaging of the anterior chamber revealed a highly significant, dose-dependent increases in chamber depth in low (Fig. 5d, g; +5.2% *p* = 0.0125), medium (Fig. 5e, h; +8.1%, *p* = 0.0447), and high (Fig. 5f, i; +24.3%, *p* = <0.0001) dose treated eyes relative to contralateral untreated control eyes (all comparisons unpaired T-tests).

### rAAV-mediated over-expression of *COX2* and *PTGFR* causes dose-dependent pigment dispersion from the iris

**Fig. 6:**
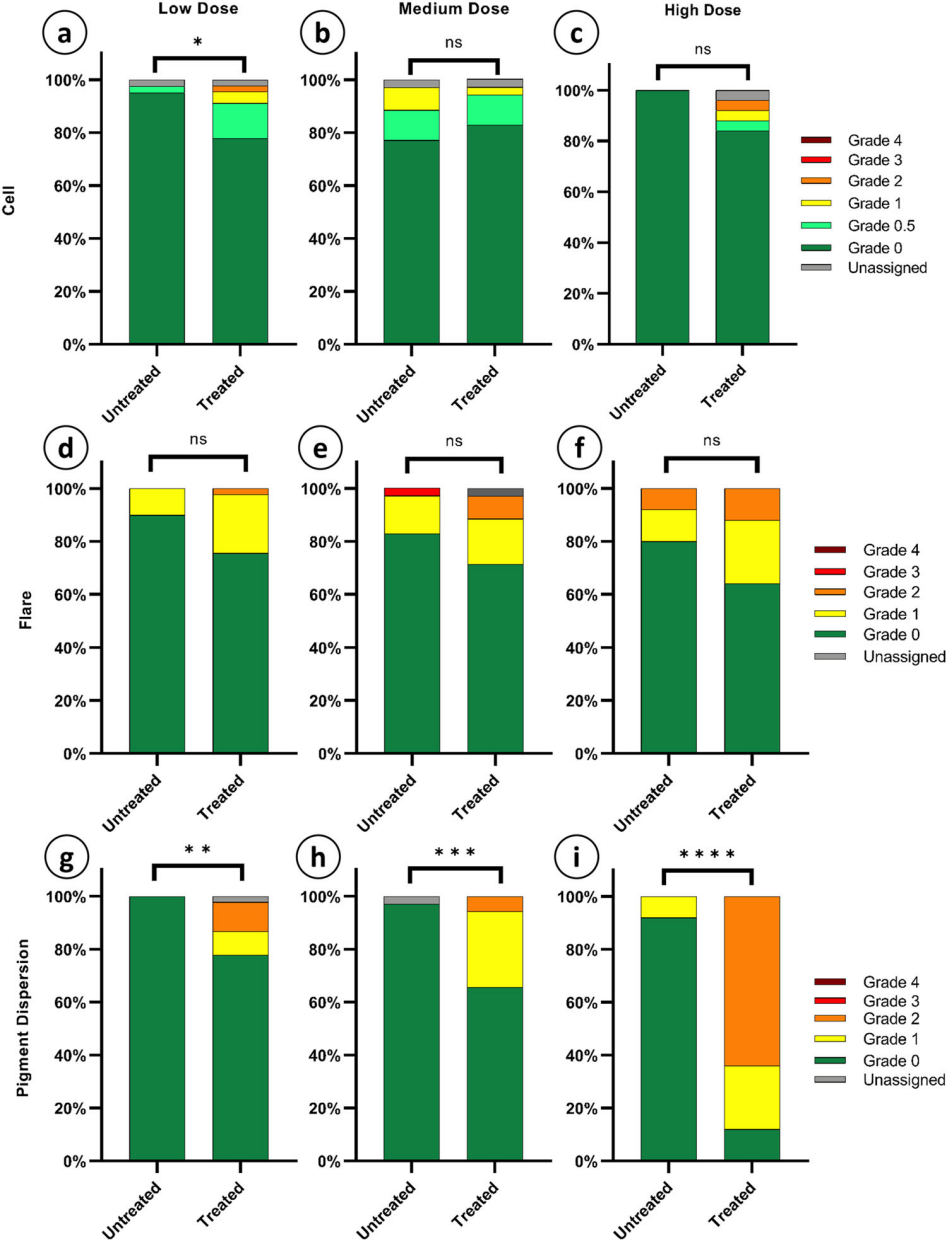
Slit lamp evaluation of treated vs. untreated eyes at 12 months. Slit lamp examination photographs graded by 5 masked participants were compared between untreated and treated eyes for each dosage group for cell (**a**–**c**), flare (**d**–**f**), and iris pigment dispersion (**g**–**i**). A Fisher’s exact test revealed that in the case of low dose cell (**a**), cell presence was dependent on vector administration, but not for medium or high dosage groups (**b**, **c**). In all cases of flare, the Fisher’s exact test revealed that flare score is independent of vector administration (**d**–**f**). Similarly, all dosage groups exhibited increased iris exfoliation that was dependent on vector administration (**g**–**i**). (Fisher’s exact test: *p*-values= 0.0165 (**a**), 0.7516 (**b**), 0.1099 (**c**), 0.0952 (**d**), 0.3938 (**e**), 0.2451 (**f**), 0.0011 (**g**), 0.002 (**h**), and <0.001 (**i**). *N* = 9, 7, and 5 for low, medium, and high dose respectively).

To determine if CCPP transgene expression or *de novo* biosynthesis of PGF_2α_ causes inflammatory responses in the anterior chamber, BN rats underwent slit lamp examinations at 12-months post-treatment looking for evidence of cell, flare, or pigment dispersion, with grading performed by 5 examiners masked with respect to both animal identity and treatment using a modified Hackett-McDonald and SUN grading scheme for uveitis^61–65^, as appropriate (Supplementary Table 1). A small, but significant increase in anterior chamber cell number was observed in low dose rAAV2/2[MAX].CCPP vector injected eyes compared to controls (Fig. 6a*p* = 0.0165, *N* = 9), but this trend was not continued in either the medium or high dose treatment groups (Fig. 6b, c, *p* = 0.7516 and 0.1099, *N* = 7 & 5 respectively). Similarly, no dosage group showed a significant association between rAAV2/2[MAX].CCPP vector treatment and the presence of flare (Fig. 6d–f; *p* = 0.0952, *p* = 0.3938, *p* = 0.3451 respectively). Conversely, pigment dispersion was observed to be significantly increased in low (Fig. 6g, *p* = 0.0011), medium (Fig. 6h, *p* = 0.0020), and high dose (Fig. 6i, *p* < 0.0001; all comparisons Fisher’s exact test) rAAV2/2[MAX].CCPP vector injected eyes. Additionally, one male and one female rat from the high dose group developed hyphema at 12 months requiring euthanasia.

**Fig. 7:**
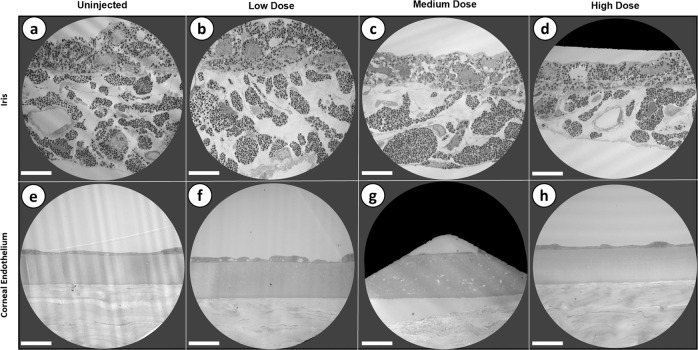
Post-mortem evaluation of tissue health through electron microscopy. Electron microscopy sections from each dosage group as well as an uninjected control eye from the high dose rat were analyzed for qualitative differences in the iris (**a**–**d**), and corneal endothelium (**e**–**h**). While the corneal endothelial cells were found to be unchanged, progressive thinning was noted in the iris as vector dosage increased. Magnification = 2000×; scale bar = 10 μm.

Electron microscopy completed post-mortem at 12-months indicate that iris morphology was found to be altered between uninjected and injected eyes, with the iris demonstrating progressive thinning as a function of increasing vector dosage (Fig. 7a–d). Corneal endothelium morphology was unchanged between the uninjected eye and injected eyes at low, medium, and high doses, indicating that CCPP expression does not adversely affect endothelial cell morphology (Fig. 7e–h).

### IOP reduction does not correlate with inflammation scoring and is not associated with ciliary body fibrosis

**Fig. 8:**
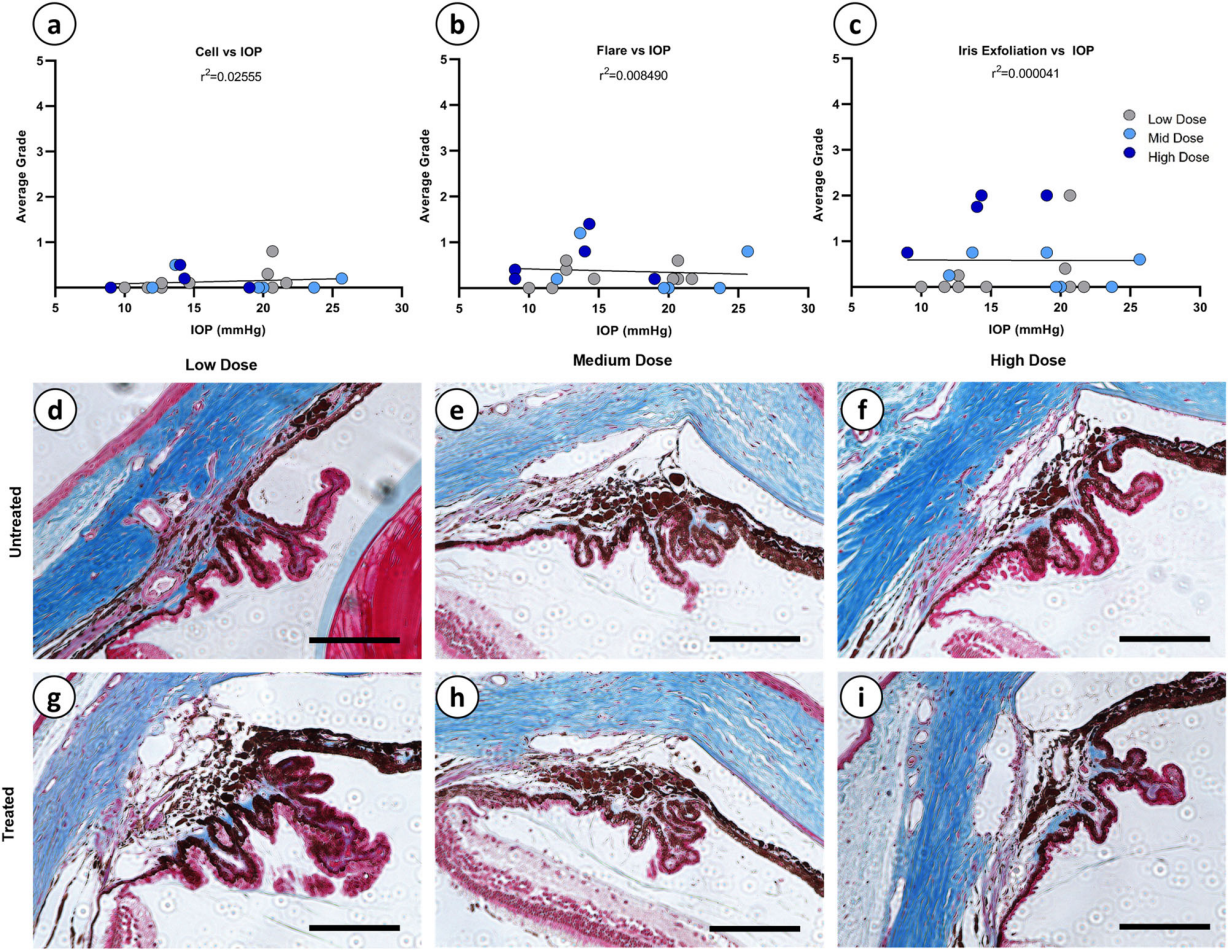
Linear regression of inflammatory responses vs IOP and Masson trichrome staining of the ciliary body. Linear regressions correlating IOP to cell, flare, and iris exfoliation (**a**–**c**) show no correlation between IOP reduction and the observed inflammation within the anterior chamber. Masson trichrome staining indicating collagen-based fibrosis (blue dye) was qualitatively analyzed in untreated (**d**–**f**) and contralateral treated eyes (**g**–**i**) (Magnification = 20×, scale bar = 150 µm), revealing no obvious differences between treated and untreated eyes at any dose.

As chronic inflammation has been associated with IOP reduction^66–68^, we sought to determine whether there was any relationship between cell, flare, or iris exfoliation scoring with measurements of IOP following rAAV2/2[MAX].CCPP vector injection across all doses. Specifically, if the reduction in IOP observed following rAAV2/2[MAX].CCPP is caused by the presence of chronic inflammation, a strong negative correlation between high inflammation scores and IOP reduction would be expected. Linear regression analyses demonstrate no significant correlation between cell (*r*^2^ = 0.02555), flare (*r*^2^ = 0.008490) or iris exfoliation (*r*^2^ = 0.000041; Fig. 8a–c). To determine whether inflammation and vector dose are covariant modifiers of IOP, we compared the slope of linear regressions for each inflammatory metric (cell, flare, and iris exfoliation) with IOP measurements and separated by vector dose (low; *N* = 9, medium; *N* = 6, or high; *N* = 5; Supplementary Fig. 4). A Tukey multiple comparison test revealed no significant difference in the slopes of regression between any doses for any inflammatory metric, including cell (*p* = 0.6316–0.6563), flare (*p* = 0.06436–0.6669) or iris exfoliation (*p* = 0.6166–0.6994), strongly indicating that ocular inflammation and rAAV2/2[MAX].CCPP vector dosing are not modifiers of IOP.

To determine whether fibrosis or atrophy of the ciliary body—which may potentially reduce aqueous humor production and so lower IOP in a therapy independent manner— was evident, immunohistochemistry was performed utilizing Masson trichrome, hematoxylin and eosin (H&E), and periodic-acid Schiff (PAS). Masson trichrome staining (Fig. 8d–i) revealed no evidence of increased fibrosis (blue dye) in rAAV2/2[MAX]. CCPP-treated eyes versus contralateral untreated control eyes at any dosage group (Fig. 8d–i). H&E (Supplementary Fig. 5) and PAS (Supplementary Fig. 6) staining revealed no obvious differences in ciliary body morphology between treated and untreated eyes at any dosage group, indicating an absence of ciliary body atrophy.

Together, this data strongly indicates that the dose-dependent reduction in IOP observed following rAAV2/2[MAX].CCPP treatment is not associated with either vector- or transgene-mediated inflammation, where there was no significant correlation between inflammatory metrics, dose, and pressure. Furthermore, there was no evidence of gross anatomical lesions (fibrosis or atrophy) affecting the ciliary body that may indicate substantial reduction of aqueous humor production.

## Discussion

Patient compliance with existing pharmacological therapies (e.g., eye drops) for open angle glaucoma is extremely poor owing to a combination of low tolerability, difficulties with self-administration, and lack of immediate negative feedback (e.g., pain) to reinforce proper dosing, leading frequently to the development of vision-threatening complications even in patients diagnosed early. In order to reduce patient non-compliance, improve visual outcomes, and lower the medical burden of adhering to a daily treatment regimen, there has consequently been substantial interest in the development of novel therapies that act to lower IOP over an extended period, including implantable drug devices and more recently, gene therapy^35,69–71^. Herein, using a combination of multimodal imaging, tonometry, electrophysiology, and post-mortem histology we demonstrate the efficacy and safety of an intracamerally injected rAAV vector which catalyzes the *de novo* biosynthesis of PGF_2α_ and its receptor from cells of the iridocorneal angle and corneal endothelium, leading to a dose-dependent, long-term (12-months) reduction in IOP. Importantly, we demonstrate that the inclusion of tetracycline-responsive riboswitch elements within the expression construct allows for IOP reduction to be temporarily reversed through supplementation of an oral activating drug—a key safety feature for clinical translation that allows either cessation of treatment in the advent of a concurrent infection (e.g., herpetic keratitis) or the ability to personalize gene dosing to the optimal transgene expression level on a patient-to-patient basis.

Our data demonstrate that dose was a factor in terms of achieving optimal IOP reduction and that critically, the degree to which intraocular pressure was reduced in medium (−21.88%) or high dose (−43.22%) groups matched or exceeded that achieved using current pharmacological approaches, where previous reports of Latanoprost usage in humans show an average reduction in IOP of 24–35% after 2 years of continuous treatment^15,72–74^. The clear dose-dependent relationship between the number of vector genomes administered and the observed reduction in IOP is of particular significance as it indicates that the proposed gene therapy treatment for OAG might be personalized based on the requirements of individual patients. Interestingly, while dosing is an important factor for achieving the desired IOP reduction, the number of vector genomes administered was also found to correlate directly with our ability to reverse the treatment effect through activation of the TC40 and TC45 tetracycline responsive riboswitch elements incorporated in the CCPP vector genome as an added safety feature^46^. When placed on a tetracycline diet to post-transcriptionally downregulate transgene expression for one month, BN rats in the medium dose group exhibited a near return to baseline, whereas high dose animals demonstrated only a partial reversion to normotension, indicating that there is a limited extent to which transgene expression, and therefore IOP reduction, can be inactivated using the current iteration of our expression cassette. One possibility for not observing full reversion to normotension in the high dose treated animals may include that sustained PGF_2α_ biosynthesis may lead to irreversible remodeling of the uveoscleral outflow pathway, although this was not immediately evident on either light or electron microscopy. An alternative explanation for the only partial reversion observed is the limited dynamic range of the tetracycline riboswitches used in this study, wherein we have previously demonstrated that the TC45 and TC40 elements have a relatively small dynamic range (~4.6-fold reduction) between inactive and active states and do not completely ‘switch off’ transgene expression, making it likely that in high dose treated eyes there was residual PGF_2α_ biosynthesis sufficient to maintain a degree of IOP reduction, even when placed on a tetracycline diet^45^. If this were demonstrated to be correct, then it would be beneficial for future studies and clinical translation to develop riboswitches with greater dynamic range and a lower expression level in the active ‘off’ state. Ideally, given the known clinical side-effects associated with long-term tetracycline usage (e.g., gut dysbiosis and tooth calcification) and the growing concerns related to antibiotic resistance, any such novel riboswitch should be designed to respond to an activating ligand that is not an antibiotic and has greater bioavailability than tetracycline, which has been shown to inefficiently cross the blood brain barrier^75,76^. Several alternative riboswitches exist, including those with greater dynamic range, including GuaM8HDV and TAP1, which are responsive to guanidine HCL and theophylline, respectively, but it is unknown whether these ligands are readily absorbed through the blood aqueous barrier^45,46,77–80^.

Our study demonstrated that the effects of CCPP transgene over-expression does not appear to negatively affect either retinal morphology or function. Although cSLO and OCT analysis revealed increased NIR and significant retinal thinning at various eccentricities from the optic nerve at 12-months compared to baseline, these changes occurred in both treated and untreated eyes and were independent of dose, where low-dose treated eyes (and contralateral controls) exhibited greater retinal thinning than those of medium or high dose treated animals. In the absence of a clear dose-dependent relationship between our CCPP therapy and increased reflectivity or retinal thinning, we believe that the observed changes are related to normal aging, where retinal thinning, especially of the retinal nerve fiber layer, has been shown previously to correlate with advancing age in humans, mouse, and rat models^54–60^. Importantly, retinal function as assessed by ffERG was not affected in any dose group under any illumination parameters. Taken together, this data suggests that intracameral administration of rAAV2/2[MAX].CCPP and *de novo* biosynthesis of PGF_2α_ is well tolerated and does not adversely affect retinal health or functionality, with untreated and treated eyes in each dose cohort showing a similar degree of increased reflectivity and retinal thinning and with no functional deficits observed by ffERG.

One of our primary considerations when evaluating the effectiveness of the proposed IOP lowering gene therapy was to assess the health of the anterior chamber following intracameral vector administration and prolonged expression of the CCPP transgene. Corneal thickness was found to be unchanged between untreated and treated eyes at 12 months, indicating that our gene therapy treatment does not adversely affect the health of the cornea and that IOP measurements taken throughout the study would not have been confounded by changes in corneal thickness in any dose group. Furthermore, EM revealed that the corneal endothelium, Descemet’s membrane, and the stroma had a normal morphology with no evidence of edema, thinning, or delamination noted, indicating that the integrity of the cornea and endothelium was not compromised. Interestingly, we did observe a significant and dose-dependent increase in anterior chamber depth, a finding in contrast to previous studies indicating that treatment with prostaglandin analogs leads to a decrease in anterior chamber depth^81,82^. However, these studies looked only at short term effects of latanoprost and pilocarpine usage in glaucomatous patients, where anterior chamber depth may be expected to be deeper than age- and gender-matched controls as a direct result of elevated IOP^82^. As a consequence, it is possible that the anterior chamber deepening observed in this study may be an artifact of using normotensive animals throughout the study, which would be expected to have normal baseline corneal curvatures and so may demonstrate pronounced alterations in chamber depth when IOP is lowered artificially via gene therapy treatment. Alternatively, it is also possible that *de novo* biosynthesis of PGF_2α_ within the anterior chamber itself leads to more extensive uveoscleral remodeling than is observed when prostaglandin analogs are applied topically to the corneal surface, as only 5–10% of each drop is taken up by the cornea and the remainder is mis-absorbed by surrounding tissues^83–86^. It remains unclear through what mechanism sustained intra-ocular biosynthesis of PGF_2α_ is affecting anterior chamber depth, however, although OCT would appear to show the central cornea of high dose eyes is more curved, indicating that the cornea has ‘bulged out’, rather than the lens and iris have moved posteriorly. In addition to the greater than desired reduction of IOP in the high dose eyes, deepening of the anterior chamber is also concerning as it would likely have an adverse impact on patient vision, where previous studies have shown that after IOL insertion in cataract patients, anterior chamber depth increases of 1 mm can cause a myopic shift in refractive errors increasing up to 0.32 diopters^87,88^.

Cell and flare scoring conducted by masked observers revealed no significant dose-dependent increase in inflammation, indicating that injection of rAAV into the anterior chamber and long-term expression of the CCPP transgene cassette is well tolerated. Conversely, while anterior chamber inflammation was found to be negligible, iris dispersion was observed in all dosage groups, with high dose animals showing highly significant levels of melanocyte migration that correlated strongly to thinning of the iris as assessed by electron microscopy. It is not possible to determine from the present study whether dose-dependent iris exfoliation resulted from the direct expression of the CCPP transgene within melanocytes, where the rAAV2/2[MAX] serotype is capable of transducing cells of the iris, or whether it is related to increasing concentrations of PGF_2α_ within the anterior chamber at higher vector doses. Regardless, the observation of dose-dependent pigment dispersion would likely contra-indicate the use of the described rAAV.CCPP gene therapy for lowering IOP in patients with congenital (e.g., Axenfeld-Rieger) or pigment dispersion glaucoma whose irises may already be unstable and degradation of which may be accelerated either through transduction of the iris with AAV or expression of the CCPP transgene and biosynthesis of PGF_2α_. Given the lack of iris involvement in the pathophysiology of ocular hypertension in OAG (as opposed to closed angle or congenital glaucoma) it would, however, likely be beneficial to investigate the selection of naturally occurring or engineered rAAV serotypes that do not transduce the iris to eliminate the possibility that direct CCPP expression causes iris exfoliation when treating OAG or pigment dispersion glaucoma.

Another major concern when evaluating the effectiveness of a gene therapy treatment that functions by modulating pathway biology to synthesize PGF_2α_ *de novo* from cells of the anterior chamber, is whether any observed reduction in IOP can be attributed not to the pharmacological action of PGF_2α_ on uveoscleral remodeling, but by chronic inflammation in response to injection of rAAV virions in the anterior chamber or subsequent expression of the therapeutic transgene. This concern is particularly relevant when evaluating a novel treatment for glaucoma, where sustained, low-grade inflammation is known to decrease IOP in human patients, and so may also be a confounding variable in experimental animal models, such as the Brown Norway rat^66–68^. By performing a series of linear regression and covariance analyses we were able to successfully demonstrate that vector dose (low, medium, or high) and the severity of inflammation (graded for cell, flare, and iris exfoliation) are *not* significant mediators of IOP reduction, strongly indicating that the observed dose-dependent lowering of IOP across treatment groups was primarily mediated by the biological action of the delivered CCPP transgene. Indeed, the only regression analysis that approached statistical significance was the relationship between iris exfoliation and IOP in the high dose treatment group (Supplementary Fig. 4I), which trends towards a positive slope with higher inflammation scoring correlated to higher IOP; the exact opposite trend of that expected if chronic inflammation was responsible for the observed IOP reduction in this study.

One limitation to this work is that untreated control eyes were contralateral to injected eyes in all dose cohorts. Specifically, studies have shown that injection of rAAV into one eye can affect the contralateral eye, as seen in pre-clinical trials for Leber’s congenital amaurosis, wherein viral DNA was found in both the injected and uninjected anterior chamber, retinae, and optic nerves of non-human primates following intravitreal vector injection^89^. This raises the possibility that the alterations in retinal reflectivity and thickness observed in our study may arise from crosstalk between the injected and contralateral uninjected eyes, rather than as a result of normal aging. We believe that this is unlikely, however, for the following reasons: (1) CCPP expression and PGF_2α_ production was not sufficient in low dose groups to alter IOP within the treated eye itself, and so it would seem illogical that such low-level activity could adversely affect the health of the retina in the contralateral uninjected eye; (2) increased retinal reflectivity and thinning were not observed to be dose-dependent in either treated or untreated eyes, which would be expected if either were attributable to the presence of vector genomes or expression of PGF_2α_ in the contralateral eye, where crosstalk would again be expected to be greatest in high dose treated BN rats; (3) previous studies have shown that the mean anterior chamber depth in BN rats is 778 ± 42.7 microns, which is similar to our 12 month findings in untreated eyes across all doses;^90^ (4) the control eye presented for the electron microscopy study was obtained from a high dose treated rat and demonstrated no adverse morphological changes (e.g., iris exfoliation/thinning), which would have been expected if crosstalk between the injected and uninjected eyes had occurred.

In conclusion, treatment of normotensive Brown Norway rats with rAAV2/2[MAX].CCPP resulted in a dose-dependent reduction in IOP, with medium dose eyes exhibiting a clinically relevant reduction in ocular hypertension without the development of adverse side-effects. Critically, IOP reduction could be reversed through administration of an oral ligand, a critical safety feature for clinical translation that allows for temporary cessation of treatment in the event of an adverse reaction or the need to resolve a commensal infection. While iris exfoliation and deepening of the anterior chamber was observed in high dose treated BN rats, these animals also exhibited greater than clinically relevant IOP reduction and as such would appear to represent an over-dosing of the rAAV2/2[MAX].CCPP vector. Future research to determine the effects of this therapy in glaucomatous animal models, as well as discovering the optimal vector capsid tropism and tunable riboswitch combination to limit the observed side-effects at high vector doses would increase translatability of the described, long-acting, single use gene therapy treatment for open angle glaucoma.

## Methods

### Animals and anesthesia

All animal experiments were approved by the Medical College of Wisconsin’s Institutional Animal Care and Use Committee and adhere to the Association for Research in Vision and Ophthalmology statement for the use of animals in ophthalmic and vision research. 6–8-week-old BN rats (*N* = 30: 15 male and 15 female) were obtained from Charles River Laboratories (BN/Crl, Wilmington, MA, USA) and housed in a 12:12 h light/dark photoperiod with diet and water provided *ad libitum*. In addition, 3 BN rats were obtained for tropism experiments. For assessments requiring general anesthesia, animals were placed in an induction chamber (VetEquip, Livermore, CA) and sedated via inhalation of isoflurane (5% induction, 1–2.5% maintenance) provided in oxygen (100%, 0.2–1 L/min). Pupil dilation was achieved through the placement of topical mydriatic eye drops containing 2.5% phenylephrine HCL and 1% tropicamide (Akorn, Lake Forest, IL, USA).

### Cell culture and ELISA

HEK293T cells (ATCC no. CRL-11268; Manassas, VA, USA) were cultured in high glucose DMEM + Glutamax (Gibco Life Technologies, Carlsbad, CA, USA) supplemented with 10% fetal bovine serum (FBS) and 1% antibiotic-antimycotic. Cells were seeded into six-well plates at a density of 3.0 × 10^5^ cells/well and then transfected using polyethylenimine (PEI) (Polysciences, #23966-100, PA, USA) at ~70% confluency with 2 µg smCBA-TC40-*COX2*-P2A-*PTGFR*-TC45 plasmid in reduced serum (2%) DMEM + Glutamax media. After 72 h, media was harvested from the wells and analyzed in duplicates using a commercial ELISA kit to quantify the levels of PGF_2α_ (Abcam, ab133056, Cambridge, United Kingdom).

### Vector production

Briefly, HEK293T cells underwent triple transfection with (1) a plasmid expressing Rep and Cap genes for the capsid mutant rAAV2/2[MAX] vector; (2) a Helper plasmid expressing adenovirus-derived genes necessary for packaging; and (3) the transgene expression plasmid (smCBA-TC40-*COX2*-P2A-*PTGFR*-TC45; for sequence information refer to patent number US20200063137A1) in equimolar ratios (1:1:1) utilizing PEI^52^. Cells were cultured in hyperflasks (Corning, Corning, NY) for 72 h post transfection in DMEM + Glutamax with 2% FBS and 1% antibiotic-antimycotic. Following 72 h, vector was purified through iodixanol density gradient centrifugation and concentration through buffer exchange, as described previously^91,92^. A total of 5 vector preparations of rAAV2/2[MAX]-smCBA-TC40-*COX2*-*PTGFR*-TC45 were then combined and concentrated through buffer exchange in a 100 kDa filter column to increase viral titers (Amicon, Darmstadt, Germany). Titers of rAAV were determined through a PicoGreen Assay (Thermo Fisher Scientific, Waltham, MA) yielding 240 μl of 3.9 × 10^13^ vg/mL (vector genomes/mL)^93^. Vectors were diluted in HBSS + 0.014% Tween-20 to provide a three-log dosage escalation for injection: 3.9 × 10^9^ vg/eye (low), 3.9 × 10^10^ vg/eye (medium), and 3.9 × 10^11^ vg/eye (high). For in vivo tropism experiments a single vector preparation of rAAV2/2[MAX]-smCBA-mCherry was prepared similarly to the process described above yielding 80 μl of 1.17 × 10^13^ vg/mL.

### Intracameral Injections

Thirty BN rats were anesthetized, and their pupils dilated before receiving a unilateral intracameral injection of rAAV2/2[MAX]-smCBA-TC40-*COX2*-P2A-*PTGFR*-TC45 at either low (3.9 × 10^9^ vg), medium (3.9 × 10^10^ vg), or high (3.9 × 10^11^ vg) dose suspended in 10 μl HBSS + 0.014% Tween 20 with a small amount of fluorescein to visualize reflux. Briefly, under an ophthalmic surgical microscope (Leica, Wetzlar, Germany) a 6 mm round glass coverslip (Fisher Scientific, Pittsburg, PA, USA) was positioned over the corneal surface and secured in position using lubricating gel (2.5% Hypromellose, Akorn, Lake Forest, IL, USA) before the tips of a 0.12 mm notched straight forceps (#0109025, Haag-Streit John Weiss, United Kingdom) were advanced posterior to the globe to secure an extraocular muscle and prevent eye rotation. A 10 mm 33-G beveled needle attached to a 100 μl Hamilton syringe was then advanced through the cornea approximately 1 mm anterior to the limbus until the needle tip was in the center of the anterior chamber. Following the injection of 10 μl purified vector the needle tip was held in position until the IOP had stabilized—indicated visually by clearing of the corneal stroma and resumption of normal retinal perfusion—to limit excessive reflux. Similarly, three BN rats underwent bilateral intracameral injections of rAAV2/2[MAX]-smCBA-mCherry for in vivo tropism experiments receiving 1.13 × 10^11^ vg each.

### Tonometry

Intraocular pressure was measured with a handheld TonoLab rebound tonometer (iCare, Oy, Finland) calibrated for use in rats at baseline and 1-, 2-, 6-, 9-, 12-, and 13-months post-injection. All readings were performed on unanesthetized animals within a defined time-period (11:00–13:00) to limit the circadian-related variability in IOP; all investigators involved with taking IOP measurements were masked with respect to vector dose. Briefly, the tonometer was secured in a horizontal position using a retort stand and the rat was positioned using gentle physical restraint so that the test eye was ~1 mm away and orientated perpendicular to the tonometer probe. Each IOP measurement is the average of three independent recordings. Following completion of the assessment, rats were rewarded with Bacon Yummies treats (Bio-Serv, Flemington, NJ, USA) to condition them to be handled without anesthesia.

### Confocal scanning laser ophthalmoscopy (cSLO)

Animals underwent preliminary and 12-month fundus assessments with a custom multiline cSLO (modified Spectralis HRA; Heidelberg Engineering, Heidelberg, Germany). After dilation of the pupils, camera alignment was facilitated through use of the near-infrared (820 nm) reflectance imaging mode, ensuring that the fundus image was evenly illuminated, and the optic disk was properly centered. Images captured are single frames.

### Slit lamp biomicroscopy

At 12-months, all animals underwent slit lamp biomicroscopy (Topcon SL-D8Z, Topcon Medical Systems, Oakland, CA, USA) with images of treated and untreated eyes captured using a digital camera (D810, Nikon, Japan). To assess for cell and flare, a slit lamp image was generated using a 1 mm slit beam that encompassed the diameter of the globe and iris exfoliation was assessed on widefield images obtained with a 20 mm beam. For grading, slit lamp images were anonymized and presented to five independent graders in randomized order to ensure the graders were masked with respect to both vector dose and animal identity. Grading was performed in adherence to the SUN grading scheme for anterior chamber cell and flare and a modified Hackett-McDonald grading scheme for iris exfoliation (Supplementary Table 1).

### Electroretinography

BN rats underwent electroretinography assessments at baseline and 12-months post-injection. Rats were dark adapted overnight, and all experimental manipulations were conducted under dim red illumination. An Espion E2 system attached to a Color Dome (Diagnosys LLC, Cambridge, UK) with Bessel filters installed for 0, 1000, and 60 Hz positioned in a Faraday cage to reduce electrical noise was utilized for recording. After anesthetization with isoflurane, rats were placed on a heated stage to maintain body temperature while Refresh lubricating eye drops were applied to the corneas to maintain hydration and provide electrical conductivity. Solid core subdermal electrodes were inserted into the scalp and haunch serving as reference and ground electrodes, respectively, while gold wire loop electrodes were placed on the cornea of both eyes. Responses were recorded after brief (4 ms) single (1 Hz) white flash stimuli over a 6-log luminance series (-4 through 0.48 log cd.s/m^2^). A- and B-wave amplitudes were measured using Espion E2 software (Diagnosys LLC, Cambridge, UK).

### Optical coherence tomography

Retinal optical coherence tomography (OCT) was completed on BN rats at baseline and 12-months. Imaging was performed using a Bioptigen Envisu R2200 Spectral Domain OCT (Leica Microsystems, Wetzlar, Germany) equipped with a rat retina specific bore. Rectangular volume scans were obtained (1000 A-scans/B-scan, 650 B-scans) of the retina and images exported to FIJI software with the OCT reader plugin installed, where 5 frames centered on the optic disk were averaged. Resulting images were then exported into OCT Reflectivity Analysis (ORA) software where measurements were recorded for retinal thickness from the NFL to the RPE at 10 longitudinal reflectivity profiles at 250-micron eccentricities from the optic disk^53^. Anterior chamber OCT was completed at 12-months on the R2200 OCT with a 12 mm telecentric bore (1000 A-scans/B-scan, 650 B-scans). Images captured were measured with calipers for central corneal thickness and anterior chamber depth in the Bioptogen InVivoVue software.

### Histology

Whole globes injected with rAAV2.2[MAX]-mCherry were enucleated and fixed overnight in 4% paraformaldehyde (PFA) at 4 °C. Eyes were then moved to a 30% sucrose solution in PBS for 24 h at 4 °C prior to being rinsed in PBS and embedded in Tissue-Tek optimal cutting temperature media (Sakura, Torrance, CA). OCT blocks were then sectioned in 14 µm increments on a Leica CM1860 cryostat (Leica, Buffalo Grove, IL) onto Fisherbrand Superfrost Plus slides and left to dry overnight. Sections were stored at −20 °C until use. Samples were thawed at room temperature for 2 h, OCT washed off in PBS, and sections counterstained with Hoechst 33342 to visualize nuclei. Sections were imaged with a Nikon Eclipse 80i confocal microscope (Nikon, Minato, Tokyo, Japan) using the 20× objective and processed as maximum intensity projections and merged in FIJI software.

Whole globes injected with the CCPP transgene vector were fixed in 4% PFA overnight and tissue dehydrated through graded ethanol, cleared with xylene, paraffin infiltrated (Sakura Tissue Tek-VIP5; automated tissue processor), and embedded into tissue blocks (Tissue Tek-TEC, embedding center). Tissue blocks were sectioned at 4 µm (Microm HM355s) and mounted on poly-L-lysine coated slides. Sections were then deparaffinized with xylene, rehydrated, and stained with hematoxylin and eosin using an automating staining platform (Sakura Prisma) and stained manually for periodic acid Schiff and Masson trichrome using a standard protocol developed by Medical College of Wisconsin’s Children’s Research Institute Histology Core.

### Electron microscopy

Whole globes from each dosage group allocated for transmission electron microscopy were enucleated and fixed in 2% paraformaldehyde, 2% glutaraldehyde in 0.1 M sodium cacodylate buffer overnight at 4 °C. Following fixation, dissection of globes to obtain samples containing the cornea, iridocorneal angle, and anterior retina were completed and tissue post-fixed in 1% osmium tetroxide on ice. Following post-fixation, tissue was dehydrated in a methanol series (50–100%) and infused with acetonitrile. Tissue samples were then embedded in 100% Embed 812 (Electron Microscopy Sciences, Hatfield, PA, USA) and baked at 60 °C overnight. Embedded samples were processed by the Medical College of Wisconsin Electron Microscopy Core, where 70 nm sections were made using a PowerTome MT-XL ultramicrotome (RMC Boeckeler, Tucson, AZ, USA), placed on 200 mesh hexagonal grids (Electron Microscopy Science) and stained with uranyl acetate and lead citrate. Stained sections were imaged on a H-600 transmission electron microscope (Hitachi High Technologies, Schaumburg, IL, USA) at 2000× magnification.

### Statistics and reproducibility

All statistical analysis was completed in GraphPad Prism 7 (GraphPad, La Jolla, CA, USA) to determine statistical significance between groups. A Shapiro–Wilk test was used to determine all data sets were normal (α = 0.05). Unpaired T-tests were used to analyze ELISA, chamber depth, and corneal thickness data. A two-way ANOVA with Sidak’s multiple comparison post hoc test determined significant differences in retinal thickness data. Multiple unpaired T-tests were used to determine differences between ERG data. A mixed effect analysis with a Dunnett’s multiple comparisons post hoc test and simple linear regression was used to analyze IOP data. Slit lamp data was analyzed using a Fisher’s exact test. To determine if a correlation between IOP reduction and inflammation was evident, simple linear regressions for inflammatory metrics vs. IOP at 12-months were completed. Additionally, to test if inflammation was a mediator of IOP reduction, linear regression slopes for each dosage group were compared with a Tukey’s multiple comparison test.

### Reporting summary

Further information on research design is available in the Nature Research Reporting Summary linked to this article.

## Supplementary information

Supplemental Information

Reporting Summary

## Data Availability

All data needed to evaluate the conclusions in the paper are present in the paper and/or Supplementary Materials. The raw data used to generate IOP, OCT, ERG, and slit lamp figures are available on Figshare at the following 10.6084/m9.figshare.21280383. Any other requests may be communicated to the corresponding author.
