# Supplementary material for: Prostaglandin-based rAAV-mediated glaucoma gene therapy in Brown Norway rats
Source: Commun Biol. 2022 Nov 3;5:1169. doi: 10.1038/s42003-022-04134-w (PMC9633612; doi:10.1038/s42003-022-04134-w)
Supplement: Supplementary file 1 — Supplemental Information [file 42003_2022_4134_MOESM1_ESM.pdf]

Prostaglandin-based rAAV-mediated glaucoma gene therapy in Brown Norway rats

Supplementary Information

Authors: Kristina J. Chern[1,2], Emily R. Nettesheim[2], Christopher A. Reid[1,2], Nathan W. Li[2], Gavin J. Marcoe[2] & Daniel M. Lipinski[1,2,*]

Affiliations:

[1] Cell Biology, Neurobiology and Anatomy, Medical College of Wisconsin; Milwaukee, WI, USA.

[2] Department of Ophthalmology and Visual Sciences, Medical College of Wisconsin; Milwaukee, WI, USA.

[*] To whom correspondence should be addressed.

*dlipinski@mcw.edu

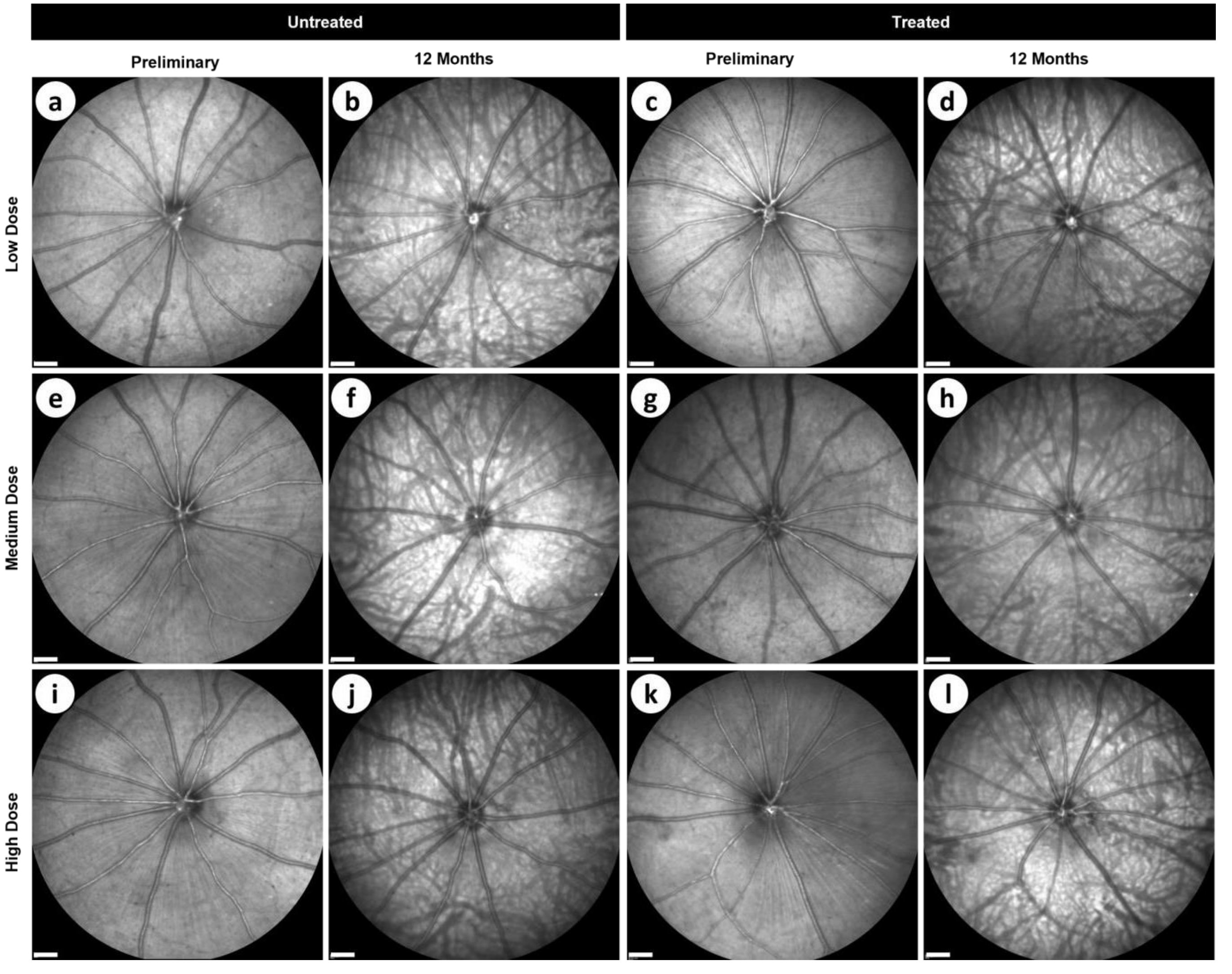

Supplementary Figure 1. **Near infrared reflectance (NIR) cSLO of untreated and treated eyes at baseline and 12 months.** Representative low dose (**a-d**), medium dose (**e-h**), and high dose (**i- l**) eyes are shown. Both untreated (**b**, **f**, **j**) and treated (**d**, **h, l**) eyes at 12 months exhibit increased NIR signal and striped fundus appearance. Scale bar = 300 µm

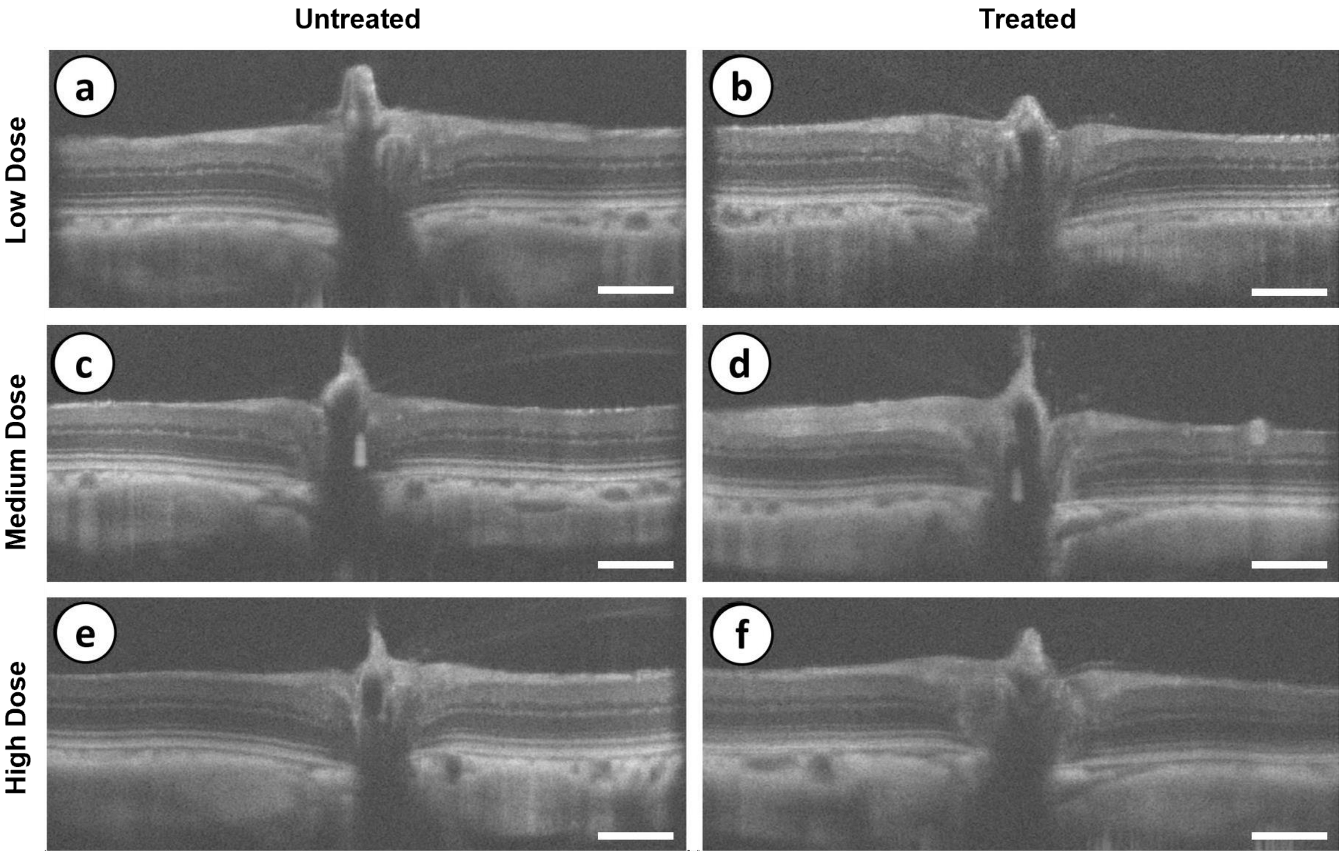

Supplementary Figure 2. Representative averaged OCT B-scans through the optic nerve head of each dosage group at 12 months. Representative untreated (**a**, **c**, **e**) and treated (**b**, **d**, **f**) OCT B-scans show no major differences between treatment or dosage groups. No evidence of inflammatory cell infiltration or retinal degeneration was found in low (**a**, **b**), medium (**c**, **d**), or high (**e**, **f**) dose treated animals in treated or contralateral control eyes. Scale bar = 200 µm

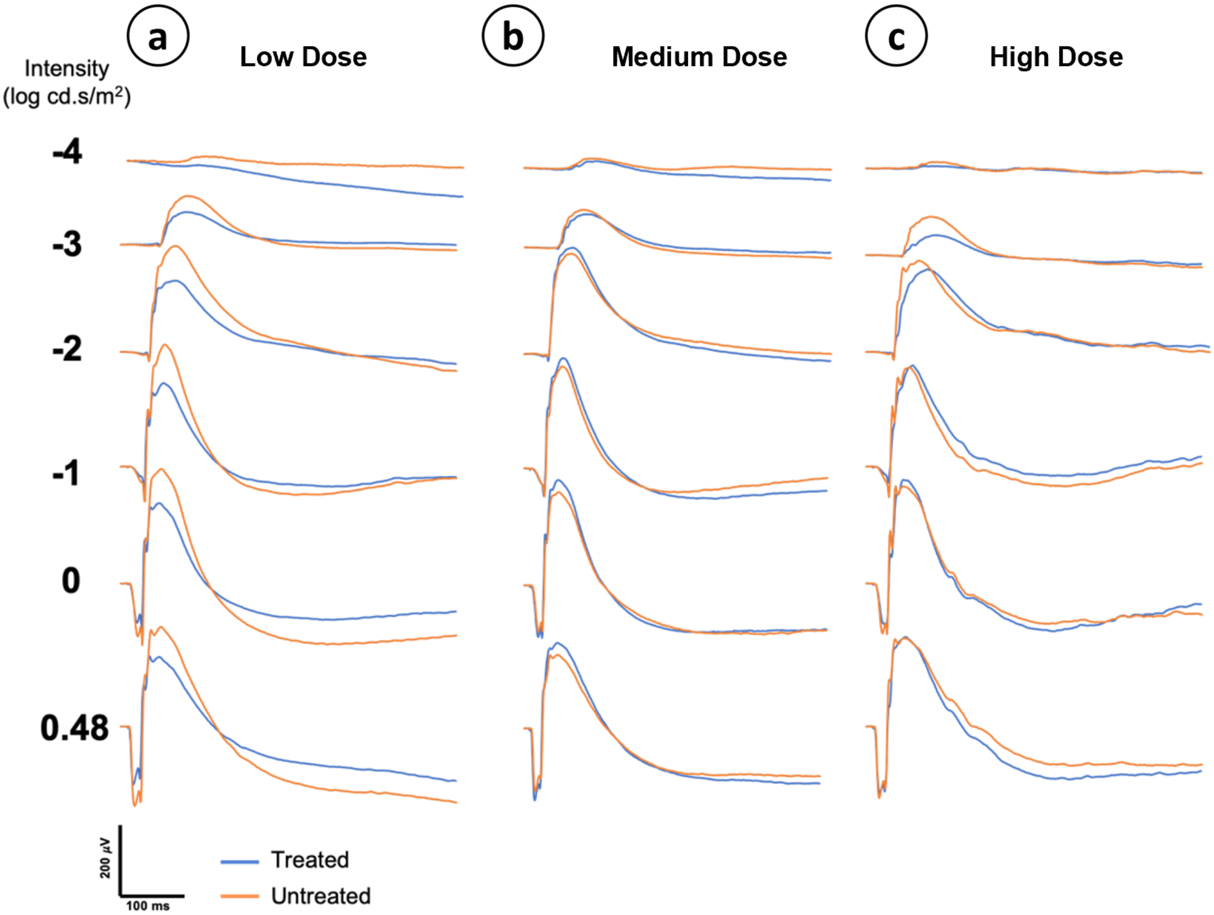

Supplementary Figure 3. Average ERG traces at 12 months. Responses were recorded and averaged from dark adapted rats after a brief (4 ms) single (1Hz) white flash stimuli over a 6-log luminance series (-4 through 0.48 log cd.s/m^2). In low dose treated animals, treated eyes showed a smaller response than untreated eyes (**a**), however medium and high dose treated animals (**b**, **c**) were found to be similar.

Grade	AC Cells	AC Flare	Pigment Dispersion
0	No cells seen	None	Normal iris pigmentation
0.5+	1-5 cells in field	N/A	N/A
1+	6-15 cells in field	Faint	Minimal- Less than 5 small clumps of pigmented material deposited on lens/cornea
2+	16-25 cells in field	Moderate- iris and lens details are clear	Mild- More than 5 clumps of pigmented material deposited but no depigmented area observed on iris
3+	26-50 cells in field	Marked- iris and lens details are hazy	Marked- iris and lens details are hazy
4+	>50 cells in field	>50 cells in field	Severe- Marked depigmentation of the iris

Supplementary Table 1: Modified standardization of uveitis nomenclature (SUN) and Hackett-McDonald grading scale for slit lamp evaluation in Brown Norway rats. The SUN scale was used to evaluate anterior chamber cell and flare using a 1mm slit lamp beam. The Hackett-McDonald grading scale for pigment dispersion was used to evaluate the severity of pigment dispersion using a 20mm slit lamp beam.

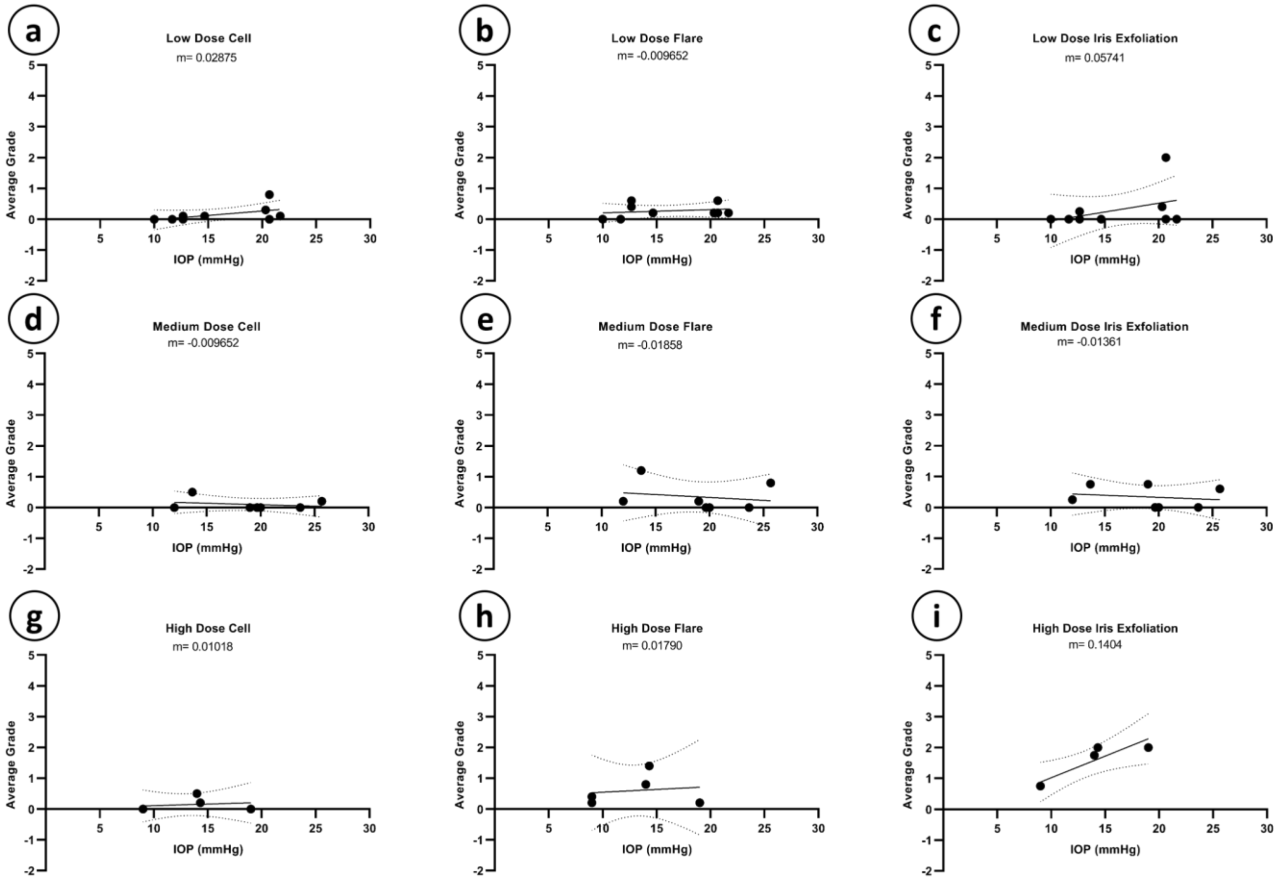

Supplementary Figure 4. Covariant analysis of linear regression slopes for inflammatory metrics in relation to IOP reduction. Grades received from 5 masked individuals were averaged for each dosage group: low (**a-c**), medium (**d-f**), and high (**g-i**). No significant correlations were found between IOP reduction and the various inflammatory parameters: cell (**a**, **d**, & **g**: p=0.6316-0.6563), flare (**b**, **e**, & **h**: p=0.06436-0.6669), or iris exfoliation (**c**, **f**, & **i**: p=0.6166- 0.6994).

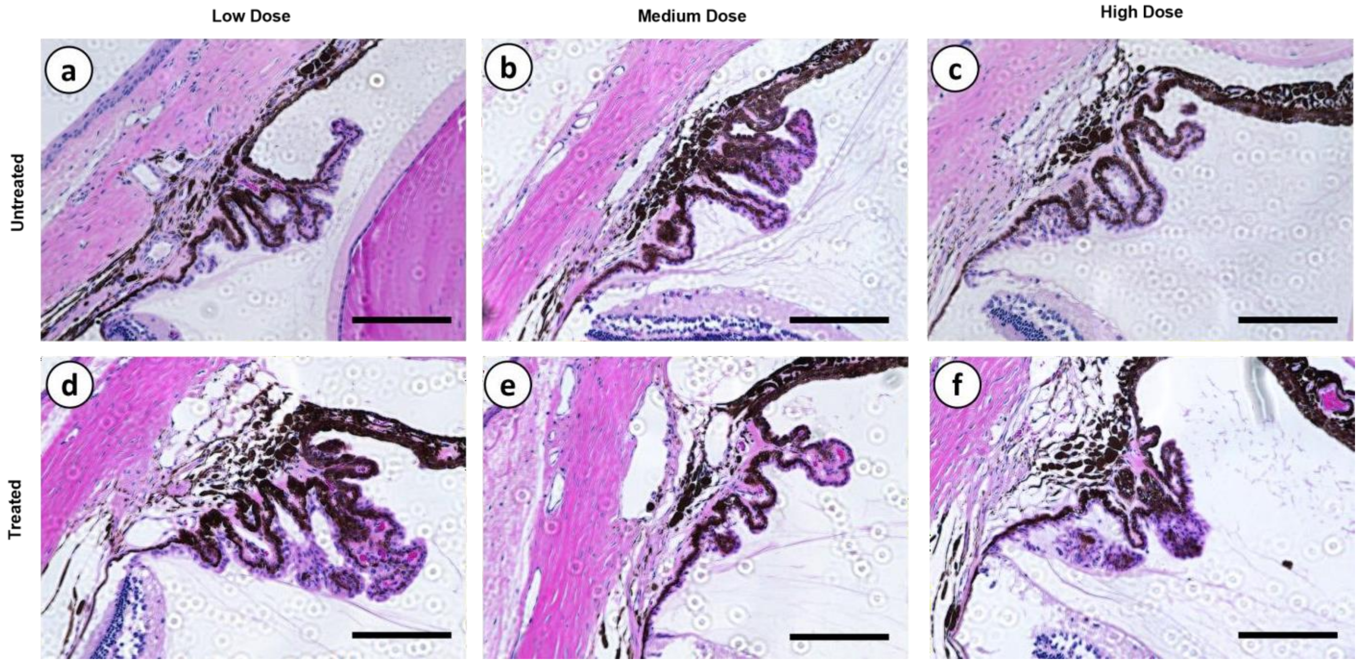

Supplementary Figure 5. Hematoxylin and eosin staining of the ciliary body in untreated vs. treated eyes. Paraffin sections from each dosage group were qualitatively analyzed for morphological changes within the ciliary body. Untreated (**a**-**c**) and contralateral treated eyes (**d**-**f**) for each dosage group show no major differences in morphology. (Magnification= 20x, Scale bar=150 µm).

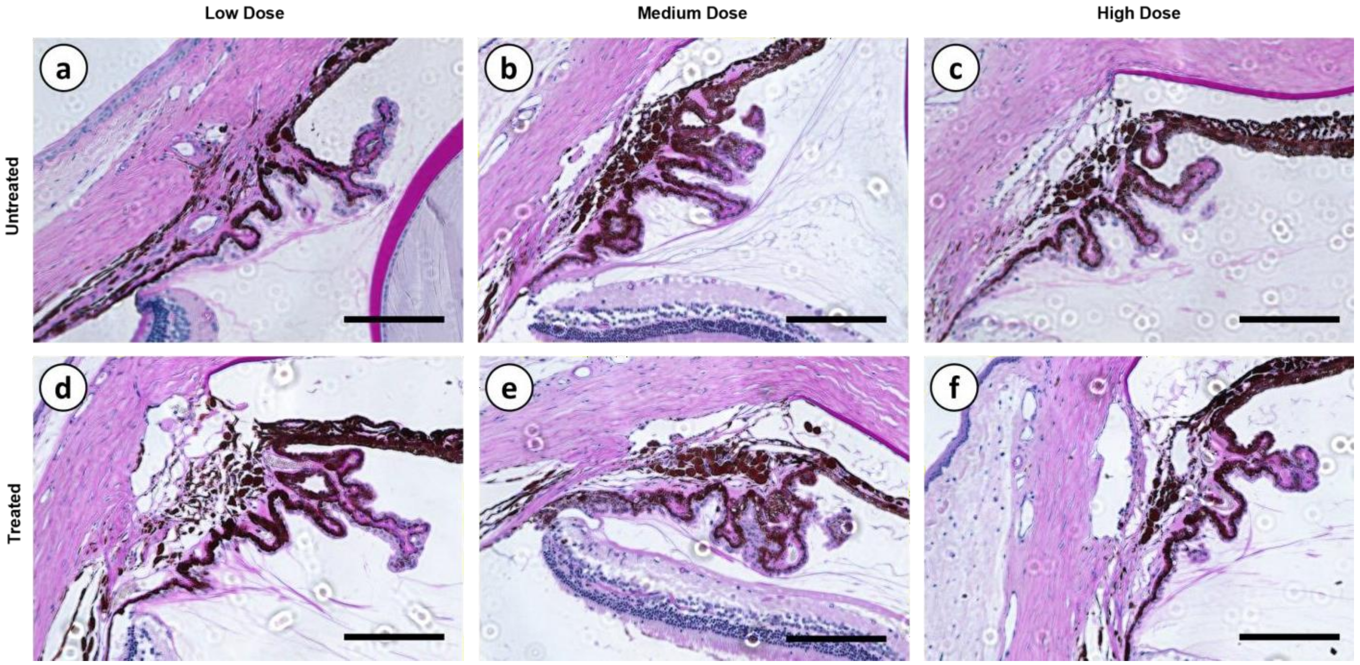

Supplementary Figure 6. Periodic acid Schiff staining of the ciliary body in untreated vs. treated eyes. PAS staining completed on paraffin sections from each dosage group were analyzed for qualitative morphological changes within the ciliary body. Untreated (**a**-**c**) and contralateral treated eyes (**d**-**f**) for each dosage group show no major differences in morphology in basement membranes, connective tissue, or glycogen deposits. (Magnification= 20x, Scale bar= 150 µm).
