# Supplementary material for: Prostaglandin-based rAAV-mediated glaucoma gene therapy in Brown Norway rats
Source: Commun Biol. 2022 Nov 3;5:1169. doi: 10.1038/s42003-022-04134-w (PMC9633612; doi:10.1038/s42003-022-04134-w)
Supplement: Supplementary file 2 — Reporting Summary [file 42003_2022_4134_MOESM2_ESM.pdf]

nature research

Corresponding author(s): Dr. Daniel Lipinski

Last updated by author(s): 10/5/2022

Reporting Summary

Nature Research wishes to improve the reproducibility of the work that we publish. This form provides structure for consistency and transparency in reporting. For further information on Nature Research policies, see our Editorial Policies and the Editorial Policy Checklist.

Statistics

For all statistical analyses, confirm that the following items are present in the figure legend, table legend, main text, or Methods section.

n/a	Confirmed	
☐	☒	The exact sample size (*n*) for each experimental group/condition, given as a discrete number and unit of measurement
☐	☒	A statement on whether measurements were taken from distinct samples or whether the same sample was measured repeatedly
☐	☒	The statistical test(s) used AND whether they are one- or two-sided<br>*Only common tests should be described solely by name; describe more complex techniques in the Methods section.*
☐	☒	A description of all covariates tested
☐	☒	A description of any assumptions or corrections, such as tests of normality and adjustment for multiple comparisons
☐	☒	A full description of the statistical parameters including central tendency (e.g. means) or other basic estimates (e.g. regression coefficient) AND variation (e.g. standard deviation) or associated estimates of uncertainty (e.g. confidence intervals)
☐	☒	For null hypothesis testing, the test statistic (e.g. F, t, r) with confidence intervals, effect sizes, degrees of freedom and P value noted<br>*Give P values as exact values whenever suitable.*
☒	☐	For Bayesian analysis, information on the choice of priors and Markov chain Monte Carlo settings
☒	☐	For hierarchical and complex designs, identification of the appropriate level for tests and full reporting of outcomes
☒	☐	Estimates of effect sizes (e.g. Cohen's *d*, Pearson's *r*), indicating how they were calculated

Our web collection on statistics for biologists contains articles on many of the points above.

Software and code

Policy information about availability of computer code

Data collection	All data was collected with commercially supplied softwares. For cSLO acquisition- Heidelberg Eye Explorer version 1.9.13.0. For OCT-InVivoVue Version 2.4.33. For ERG- Espion V6 version V6.63.26. For confocal microscopy- NIS-Elements Version 4.20
Data analysis	Confocal microscopy image merging: FIJI version V1.53o. Retinal thickness calculations- OCT Reflectivity Analytics version 1.1.0 made by Dr. Joseph Carroll's laboratory (Wilk, M. A., Wilk, B. M., Langlo, C. S., Cooper, R. F. & Carroll, J. Evaluating outer segment length as a surrogate measure of peak foveal cone density. Vision Res. 130, 57–66 (2017))

For manuscripts utilizing custom algorithms or software that are central to the research but not yet described in published literature, software must be made available to editors and reviewers. We strongly encourage code deposition in a community repository (e.g. GitHub). See the Nature Research guidelines for submitting code & software for further information.

Data

Policy information about availability of data

All manuscripts must include a data availability statement. This statement should provide the following information, where applicable:

- Accession codes, unique identifiers, or web links for publicly available datasets
- A list of figures that have associated raw data
- A description of any restrictions on data availability

All data needed to evaluate the conclusions in this manuscript are present in the paper and/or supplementary materials

Field-specific reporting

Please select the one below that is the best fit for your research. If you are not sure, read the appropriate sections before making your selection.

☒ Life sciences ☐ Behavioural & social sciences ☐ Ecological, evolutionary & environmental sciences

For a reference copy of the document with all sections, see nature.com/documents/nr-reporting-summary-flat.pdf

Life sciences study design

All studies must disclose on these points even when the disclosure is negative.

Sample size	30 brown Norway rats, 15 male and 15 female, were chosen for evaluation: 11 were used in the low dose group, 9 in the medium dose groups, and 10 in the high dose group. No sample size calculations were completed. Animal numbers were chosen to maintain feasibility for the researchers completing evaluations at each timepoint
Data exclusions	No data was excluded from this manuscript. 2 high dose treated rats were euthanized at 12 months due to developing complications (hyphema) as listed in the manuscript. 1 medium dose rat also died after complications with anesthesia at 12 months, however all data collected up to that point was included in analysis.
Replication	Data was not replicated in this study due to the long experimental timeline of 12 months.
Randomization	Animals were chosen for injection through a randomized process wherein the animal anesthesiologist chose the order of the rats to be injected, while the investigator injecting vector decided which dosage that animal was to receive.
Blinding	Investigators were blinded during IOP measurement aquisition, as well as during grading of cell, flare, and iris exfoliation. Investigators did not know the injection status of each animal while obtaining IOP measurements. Grading of cell, flare and iris exfoliation was completed on a randomized slideshow consisting of both injected and uninjected eyes wherein graders did not know the injection status of the animal

Reporting for specific materials, systems and methods

We require information from authors about some types of materials, experimental systems and methods used in many studies. Here, indicate whether each material, system or method listed is relevant to your study. If you are not sure if a list item applies to your research, read the appropriate section before selecting a response.

Materials & experimental systems

n/a	Involved in the study	
☒	☐	Antibodies
☐	☒	Eukaryotic cell lines
☒	☐	Palaeontology and archaeology
☐	☒	Animals and other organisms
☒	☐	Human research participants
☒	☐	Clinical data
☒	☐	Dual use research of concern

Methods

n/a	Involved in the study	
☒	☐	ChIP-seq
☒	☐	Flow cytometry
☒	☐	MRI-based neuroimaging

Eukaryotic cell lines

Policy information about cell lines

Cell line source(s)	HEK293T cells: ATCC no. CRL-11268
Authentication	The cell line used was not authenticated
Mycoplasma contamination	Cell lines used were not tested for mycoplasma contamination
Commonly misidentified lines (See ICLAC register)	*Name any commonly misidentified cell lines used in the study and provide a rationale for their use.*

Animals and other organisms

Policy information about studies involving animals; ARRIVE guidelines recommended for reporting animal research

Laboratory animals	6-8 week old Brown Norway rats from Charles River Laboratories (BN/Crl) were used in the study, 15 male and 15 female rats were used for a total of 30 rats
Wild animals	This study did not use wild animals
Field-collected samples	This study did not used field-collected samples
Ethics oversight	The animal usage in this manuscript was approved by the Medical College of Wisconsin's Institutional Animal Care and Use Committee and adhere to the Association for Research in Vision and Ophthalmology's statement for the use of animals in ophthalmic and vision research.

Note that full information on the approval of the study protocol must also be provided in the manuscript.
